# Integrative Proteomics and Transcriptomics Profiles of the Oviduct Reveal the Prolificacy-Related Candidate Biomarkers of Goats (*Capra hircus*) in Estrous Periods

**DOI:** 10.3390/ijms232314888

**Published:** 2022-11-28

**Authors:** Zhipeng Sun, Yufang Liu, Xiaoyun He, Ran Di, Xiangyu Wang, Chunhuan Ren, Zijun Zhang, Mingxing Chu

**Affiliations:** 1 Key Laboratory of Animal Genetics, Breeding and Reproduction of Ministry of Agriculture and Rural Affairs, Institute of Animal Science, Chinese Academy of Agricultural Sciences, Beijing 100193, China; 2 College of Animal Science and Technology, Anhui Agricultural University, Hefei 230036, China

**Keywords:** oviduct, DIA proteome, WGCNA, transcriptome, goat, estrous period, fecundity

## Abstract

The oviduct is a dynamic reproductive organ for mammalian reproduction and is required for gamete storage, maturation, fertilization, and early embryonic development, and it directly affects fecundity. However, the molecular regulation of prolificacy occurring in estrous periods remain poorly understood. This study aims to gain a better understanding of the genes involved in regulating goat fecundity in the proteome and transcriptome levels of the oviducts. Twenty female Yunshang black goats (between 2 and 3 years old, weight 52.22 ± 0.43 kg) were divided into high- and low-fecundity groups in the follicular (FH and FL, five individuals per group) and luteal (LH and LL, five individuals per group) phases, respectively. The DIA-based high-resolution mass spectrometry (MS) method was used to quantify proteins in twenty oviducts. A total of 5409 proteins were quantified, and Weighted gene co-expression network analysis (WGCNA) determined that the tan module was highly associated with the high-fecundity trait in the luteal phase, and identified NUP107, ANXA11, COX2, AKP13, and ITF140 as hub proteins. Subsequently, 98 and 167 differentially abundant proteins (DAPs) were identified in the FH vs. FL and LH vs. LL comparison groups, respectively. Parallel reaction monitoring (PRM) was used to validate the results of the proteomics data, and the hub proteins were analyzed with Western blot (WB). In addition, biological adhesion and transporter activity processes were associated with oviductal function, and several proteins that play roles in oviductal communication with gametes or embryos were identified, including CAMSAP3, ITGAM, SYVN1, EMG1, ND5, RING1, CBS, PES1, ELP3, SEC24C, SPP1, and HSPA8. Correlation analysis of proteomics and transcriptomic revealed that the DAPs and differentially expressed genes (DEGs) are commonly involved in the metabolic processes at the follicular phase; they may prepare the oviductal microenvironment for gamete reception; and the MAP kinase activity, estrogen receptor binding, and angiotensin receptor binding terms were enriched in the luteal phase, which may be actively involved in reproductive processes. By generating the proteome data of the oviduct at two critical phases and integrating transcriptome analysis, we uncovered novel aspects of oviductal gene regulation of fecundity and provided a reference for other mammals.

## 1. Introduction

Goats (*Capra hircus*) have been raised for a long time to satisfy the human requirements for meat, and with the rapid development of the China economy, goat meat consumption has been gradually increasing [1]. High fecundity in goats is a desirable and genetically correlated trait in mutton production. Many factors that affect fertility, of which the maternal environment is an important cause [2]. Previous work has started to show that maternal interactions with gametes and embryos are fundamental to pregnancy and influence embryonic development, implantation, and gestation maintenance [3]. As an important maternal organ, the oviduct is the site of final sperm capacitation and oocyte fertilization; in mammals, the zygote undergoes early development, giving rise to the morula and paving the way for the oocyte-to-embryo transition [4,5,6]. Defects in the maternal environment of the oviduct are linked to mammalian fecundity. For example, in vitro studies and somatic cell nuclear transfer has found that early embryonic development can be achieved; nevertheless, it is no substitute for the fine regulation of the oviduct throughout embryonic development [7,8]. In addition, oviduct cells always provide requirements for developing preimplantation embryos by producing embryonic trophic factors [9]. The follicular and luteal phases are the two main stages of the mammalian reproductive cycle [10]. However, the oviduct function in different phases is related to distinct metabolic, secretory, and embryo/gamete needs [11,12]. Studies of oviductal epithelial cells (OECs) in pigs have revealed that proteins associated with the follicular phase may regulate gamete function and sperm storage in the oviduct, while proteins found in the luteal phase are more likely to be involved in providing embryonic support [13]. Therefore, understanding how the oviduct affects the life activities of gamete and embryo at the molecular level is of scientific importance for elucidating the molecular mechanism of high fecundity in goats.

Gradually, more proteome-level works have been performed to reveal the molecular basis of mammalian reproduction due to the advancement of mass spectrometry (MS) techniques. Accumulating evidence suggests that proteomic analysis of oviduct activity has been performed to identify DAPs in some domestic animals. In sheep, melatonin receptors MT1 and MT2 [14] and two isoforms of dihydrotestosterone (DHT) sythetase enzymes 5α-reductase (5α-red1, 5α-red2) [15] are mainly expressed in the epithelial cells of oviduct ampulla, both of which are regulated by estradiol (E2) via the estrogen receptor (ER) pathway and affect the function of the oviduct membrane and sperm–oocyte binding. Proteomic analysis of oviduct cells in cattle has reported that heat shock protein 90 beta family member 1 (HSP90B1), oviductal glycoprotein 1 (OVGP1), and glutathione peroxidase 4 (GPX4) were upregulated in the follicular phase [16]. Among themes, HSP90B1 and that GPX4 play a role in embryo stress protection [4,17], and OVGP1 interacts with the oocyte, sperm, and embryo [17]. Furthermore, motility modulation has also been observed in spermatozoa exposed to oviductal fluid [18] or proteomic components of OEC [19], emphasizing the role of oviductal proteins in mediating gamete function. In vitro culture experiments of goat embryos showed that adding purified, oviduct-specific, estrus-inducing glycoprotein to the culture medium could significantly improve the blastocyst development rate [20].

Most investigations in the regulation of reproduction focus on transcriptome profiling because it is sensitive and cost-effective [21]. However, studies have shown that mRNA may not be a reliable proxy for protein expression [22,23]. Recent advances in multi-omics have facilitated the generation of large datasets that can be used to investigate complex life activities [24,25]. These enable us to precisely determine how mRNAs [26] and proteins [27] are organized into network structures. Multi-omics approaches can potentially to improve understanding of cellular life processes by elucidating them from multiple perspectives. Comprehensive proteomic and transcriptomic analyses of the oviduct in the follicular and luteal phases, on the other hand, are largely unknown and warrant further investigation.

The characteristics of high fecundity and perennial estrus in Yunshang black goat are of great value to understanding goat reproduction mechanisms [28]. In this study, an effective proteomic method, data-independent acquisition (DIA), was applied to twenty oviducts to identify protein profiles. These DAPs highlighted the oviduct functions, laying the foundation for an in-depth study on the regulatory mechanisms of goat kidding numbers. WB confirmed the expression of the hub proteins in high- and low-fecundity groups at different phases. Moreover, we examined oviductal mRNA levels during the same period. The abundance of transcripts and proteins for most genes expressed in the oviduct was driven by the estrus stage. This provides further insight into characterizing the relationship between mRNA expression and protein levels for goat fecundity in the oviduct. In summary, we investigated the molecular mechanism of the oviduct affecting the kidding number in goats by combining proteomic and transcriptomic techniques, which revealed the molecular functions of the oviduct under different estrous cycles, as well as the key components regulating fecundity in goats.

## 2. Results

### 2.1. Overview of Quantitative Proteomics Analysis of Goat Oviducts

**Figure 1:**
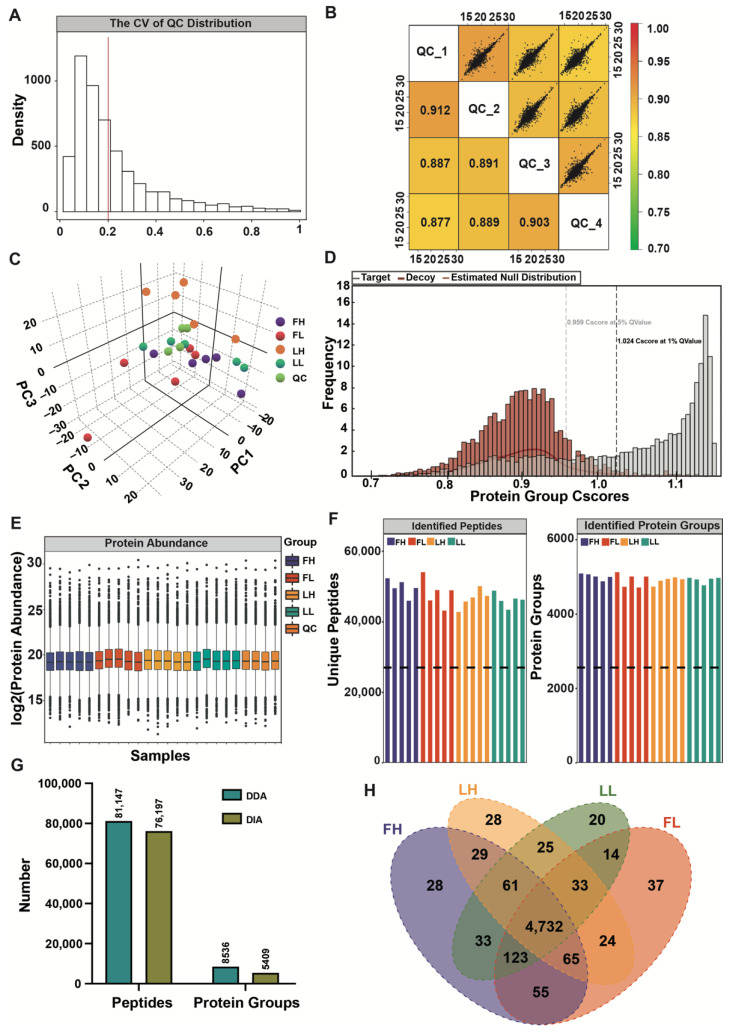
Overview of quality control (QC) and quantitative proteomics. (**A**) The coefficient of variation (CV) of QC distribution. (**B**) Correlation analysis of QC samples; the correlation coefficient close to 1 indicates that the system is more stable. (**C**) 3D Principal component analysis (PCA) of the quantified proteins for each QC sample. (**D**) Protein FDR analysis; the 1% Q Value is used as the qualitative threshold, which corresponds to FDR 0.01, red is the distribution of decoy (anti-library), gray is the target (positive library), C-score means the protein confidence score, and a higher C-score at 1% Q Value indicates a better result. (**E**) The quantitative intensity distribution of all samples and QC samples. (**F**) Statistics of the peptides and proteins identified in each sample; the dotted line represents the number of proteins or peptides at 50% of the total maximum number of identifications. (**G**) Comparison of peptides and proteins in data-dependent acquisition (DDA) and data-independent acquisition (DIA) strategies. (**H**) Venn diagram of proteins in different groups.

The median intra-group coefficient of variation (CV) distribution for the four-quality control (QC) replicates was around 20% (Figure 1A). The Pearson correlation coefficient at the protein level between every two QC samples indicated that the replicates of the four QC samples were highly correlated, with a Pearson correlation coefficient of approximately 0.9 (Figure 1B). Principal component analysis (PCA) showed that samples from four different groups and QC samples could be separated, and samples from the same stage clustered well (Figure 1C). The DIA-based quality control is shown in Supplementary Figure S2. The false-discovery rate (FDR) method was applied to conduct multiple testing corrections. A *q* value of 0.01 was set as the threshold, which was equivalent to FDR 0.01. In this study, the C-score was 1.024 with a *q* value of <0.01, indicating that the qualitative results are highly reliable (Figure 1D). Furthermore, we counted the protein expression abundance of the twenty samples, and the results showed that the protein expression was relatively consistent among the five biological replicates in different groups (Figure 1E). These results confirmed the high degree of reproducibility of MS data acquired by the DIA method.

In this study, each sample was quantified, and we selected proteins at constant levels found in more than 50% of samples for follow-up statistics (Figure 1F, Supplementary Table S1). In total, 81,147 peptides and 8536 proteins were quantified in the data-dependent acquisition (DDA) spectral library (Figure 1G). The DIA data with the DDA spectra library were analyzed by Spectronaut Pulsar X; about 76,197 peptides and 5409 proteins were further quantified (Figure 1G, Supplementary Data S1). The quantitative heat map of the total DIA-identified proteins from all samples is shown in Supplementary Figure S2D. In addition, we statistically counted the overlap of proteins between different groups, and 4732 proteins overlapped in the four groups (Figure 1H).

### 2.2. Oviduct Protein Co-Expression Network Analysis Identifies Module–Trait Relationships and Points to Core Mediators of Fecundity

**Figure 2:**
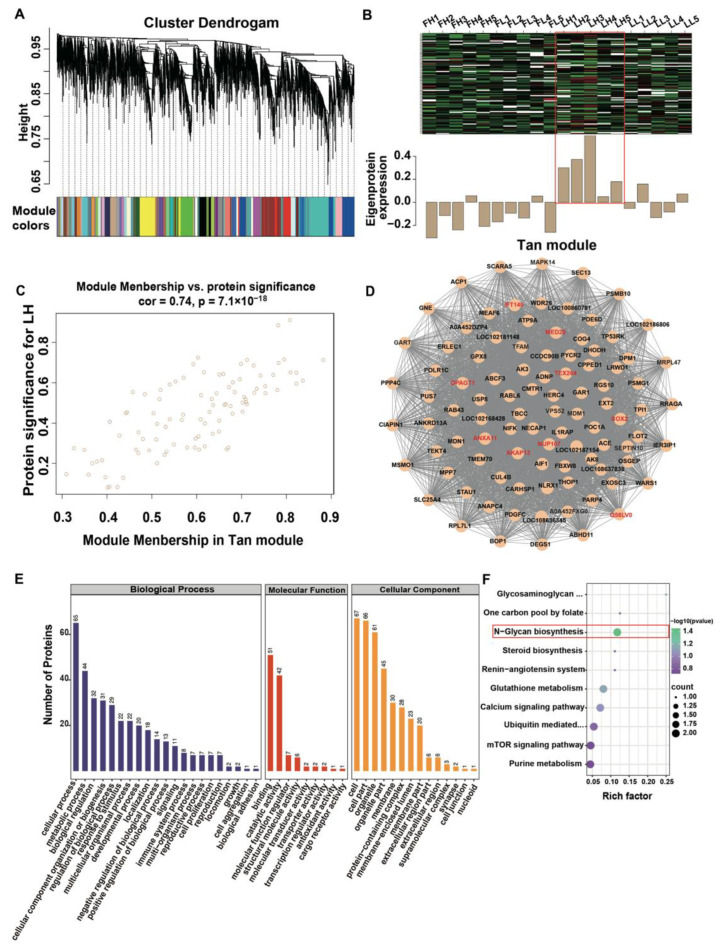
Protein co-expression network analysis of the oviduct proteome identified modules associated with high fecundity. (**A**) Module colors represent the final modules. Each branch in the hierarchical tree or each vertical line in the color bars represents one protein. (**B**) Eigengene expression pattern of the tan module. (**C**) A scatterplot of proteins significance for high fecundity in the luteal phase (LH) vs. module membership in the tan module. (**D**) The co-expressed protein network of the tan module. (**E**) GO analysis of Tan module proteins. The vertical coordinate indicates the GO Level 2 annotation information, including Biological Process, Molecular Function, and Cellular Component; the horizontal coordinate indicates the number of DAPs under each functional category. (**F**) KEGG enrichment analysis of Tan module proteins. Only the N-Glycan biosynthesis pathway was significantly enriched with a *p* value < 0.05.

In total, 5076 proteins were screened from the 5409 to reveal the expression patterns of proteins inside various WGCNA modules following the determination of a soft threshold at R^2^ = 0.9 (Supplementary Data S2). Thereafter, 45 modules were identified through the average linkage hierarchical clustering in the four groups and are visualized in Figure 2A (Supplementary Data S2). The co-expressed protein heat map revealed that proteins in the same model had a high biological relevance (Figure S3A). To assess the significance of the modules with prolificacy traits, we correlated the modules with fecundity hallmarks of goats in the different estrus cycles (Figure S3B; Supplementary Data S2). In the follicular phase, the blue module (475 proteins, cor = −0.45, and *p* = 0.04) negatively correlated with the trait of high fecundity (FH); light-yellow, brown, and magenta modules (69, 385, and 119 proteins, respectively; cor = 0.5, and *p* = 0.02) showed the highest correlation with low fecundity (FL). In the luteal phase, the tan module (96 proteins, cor = 0.76, and *p* = 9 × 10^−5^ and steel-blue module (49 proteins, cor = 0.55, and *p* = 0.01) showed the strongest correlation with the trait of high (LH) and low (LL) fecundity, respectively. These modules suggest that the proteins in the modules were significantly associated with the corresponding trait. Of note, the tan module had a significantly positive correlation with the high-fecundity trait in the luteal phase and was selected for further analysis (*p* < 0.05) (Figure 2B, Supplementary Figure S3B). The scatter plot demonstrated that the significance and module membership of the correlated proteins in the tan modules were significantly associated with LH, as shown in Figure 2C.

We built a protein-protein interaction (PPI) network using Cytoscape software (V.3.9.0) for the tan module with several hub proteins with the most connectivity (Figure 2D, Supplementary Data S2). Some of the top hub proteins in this module are essential for cell migration; germ-cell and embryo development; reproductive hormone secretion; and oviduct function, including NUP107, ANXA11, TEX264, DPAGT1, MED20, COX2, AKAP13, IFT140, and Q58LV0 (somatotropin), which may create an optimal intrafallopian environment for early embryo development. In addition, the gene ontology (GO) databases were used to analyze the functional of tan module proteins based on three categories, including biological process (GO BP), cellular component (GO CC), and molecular function (GO MF) (Figure 2E, Supplementary Data S3). In the GO BP category, reproduction and biological adhesion were associated with oviduct functions. Binding, catalytic activity, and the molecular function regulator were the three groups that were most prevalent in GO MF. The most prevalent GO CC categories were related to cells, cell parts, organelles, and membranes. Mitogen-activated protein kinase (MAPK14), POC1 centriolar protein A (POC1A), angiotensin-converting enzyme (ACE), nuclear pore complex protein (NUP107), tektin 4 (TEKT4), F-box and WD repeat domain containing 8 (FBXW8), and NAD(P) dependent steroid dehydrogenase-like (NSDHL) were enriched in the reproductive process, reproduction, and development process, which may be significantly associated with the fecundity of the goat. As for the KEGG enrichment analysis, just the N-Glycan biosynthesis pathway was significantly enrichened (*p* < 0.05) (Figure 2F, Supplementary Data S3), and GlcNAc-1-P transferase (DPAGT1) and dolichol-phosphate mannosyltransferase subunit 1 (DPM1) participated in this pathway.

### 2.3. Identification of Differentially Abundant Proteins

**Figure 3:**
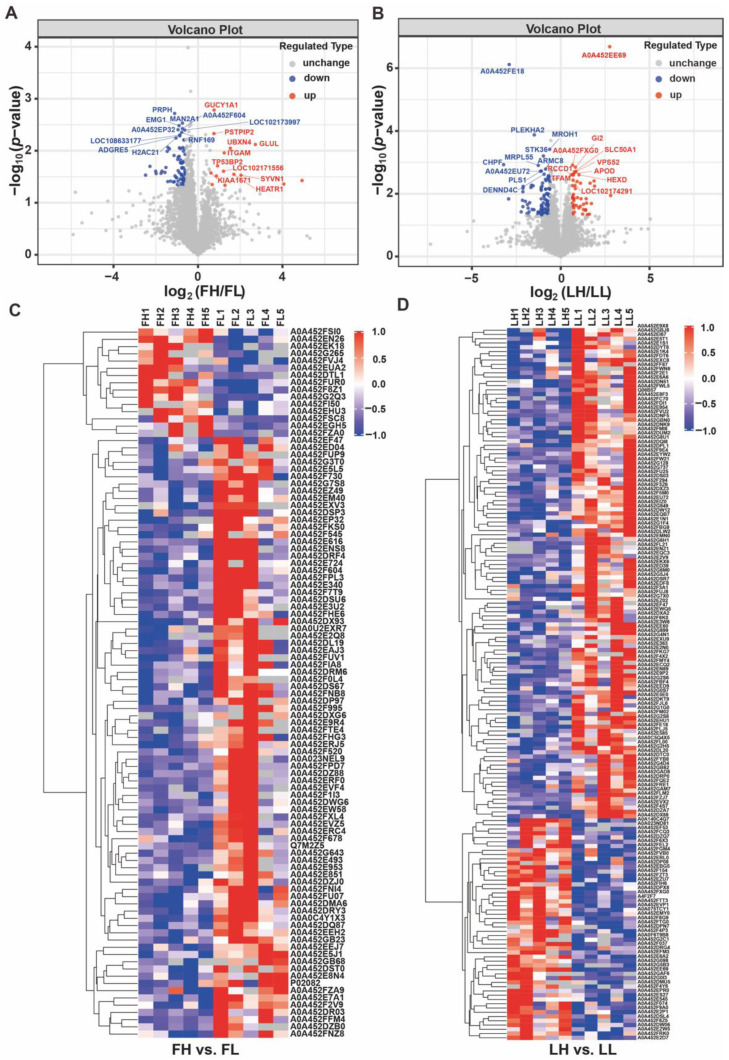
Differential abundant proteins (DAPs) screening and clustering analysis. Volcano plot of DAPs in the two comparison groups of FH vs. FL (**A**) and LH vs. LL (**B**). The X-axis represents the fold change in protein expression of different comparison groups. The Y axis represents the statistical significance of proteins. The blue dots represent the proteins that were not significantly downregulated, the red dots represent proteins that were significantly upregulated, and the gray dots represent proteins that were not significantly different. Cluster analysis of DAPs in the group of FH vs. FL (**C**) and LH vs. LL (**D**). The hierarchical clustering results are represented as a tree heat map, with the ordinate representing significantly differentially abundant proteins and the abscissa representing sample information. Red represents significantly upregulated proteins, blue represents significantly downregulated proteins, and gray represents no quantitative information for proteins.

**Figure 4:**
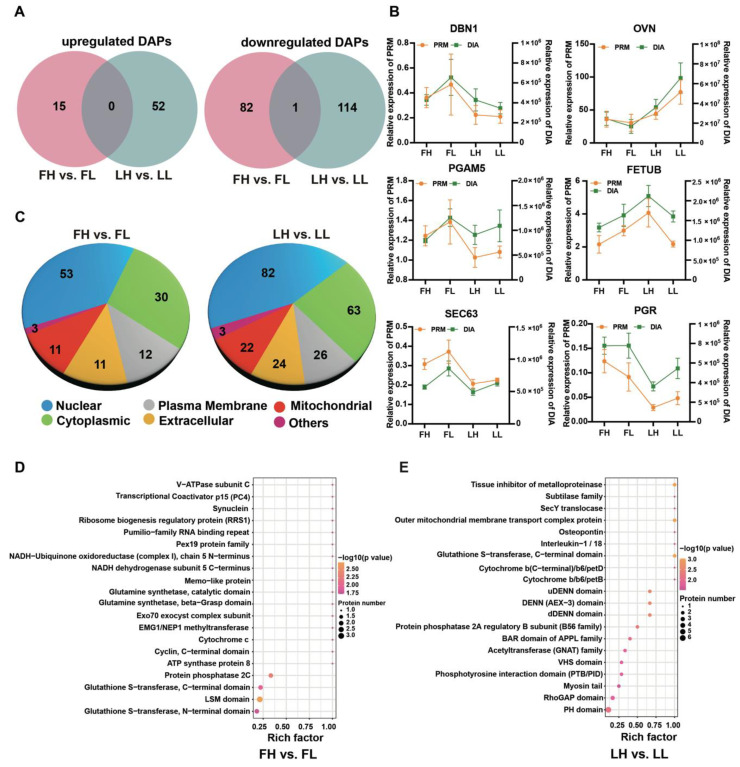
Functional annotation analysis of differentially abundant proteins (DAPs). (**A**) Venn diagram of DAPs identified between the follicular phase and luteal phase in the oviduct. (**B**) Validation results of six selected proteins in the four groups (FH, FL, LH, and LL) by parallel reaction monitoring (PRM). (**C**) Subcellular localization of DAPs in the different comparison groups. (**D**,**E**) Domain enrichment analysis of DAPs in the FH vs. FL (**D**) and LH vs. LL (**E**) comparison groups.

A total of 4975 and 4851 proteins were identified in the FH vs. FL and LH vs. LL comparisons, respectively (Table S1), while 98 and 167 DAPs were identified in each comparison group. In the FH vs. FL comparison group, 15 of these DAPs showed upregulation, 83 showed downregulation (Figure 3A), and 52 showed upregulation; 115 showed downregulation in the LF vs. LL comparison group (Figure 3B). DAPs were further selected to build a heatmap (Figure 3C,D), which revealed the expression patterns in the follicular and luteal phases of goat oviducts. Moreover, the similarity of data patterns within each group is high, while it is low between groups, which effectively differentiates the groups. The Venn diagram of the DAPs showed that just one downregulated DAP was co-expressed between the two phases (Figure 4A). These results suggested that the follicular phase of the oviduct and luteal phase have different protein profiles in response to the prolificacy trait of goats. 

There are a few key DAPs that enriched in the follicular phase (Supplementary Data S1), demonstrating the important roles the oviduct plays. For example, proteins involved in the energy processes (ATP synthase protein 8, ATP8; ATPase H+ transporting V1 subunit C1, ATP6V1C1; ATP synthase membrane subunit f, ATP5MF; mannosidase alpha class 2A member 1, MAN2A1), the cellular adhesion and transporters (adhesion G protein-coupled receptor E5, ADGRE5; actin filament associated protein 1 like 2, AFAP1L2; mediator of cell motility 1, MEMO1), and the calcium regulatory (calmodulin-regulated spectrin-associated protein family member 3, CAMSAP3; calcium/calmodulin-dependent serine protein kinase, CASK). We also found that reproductive-related proteins, such as TAB1 (TGF-beta activated kinase 1 (MAP3K7) binding protein 1), were highly enriched in the FH vs. FL comparison group. For the luteal phase, such as cytoskeleton (cytoskeleton-associated protein 5, CKAP5; SWI/SNF related, matrix associated, actin dependent regulator of chromatin, subfamily a, member 1, SMARCA1) and metabolic transporters (sphingolipid transporter 1, SPNS1; solute carrier family 50 members 1, SLC50A1; actin-related protein 2/3 complex subunit 3, ARPC3; transmembrane p24 trafficking protein family member 8, TEMD8; trafficking protein particle complex subunit 5, TRAPPC5; target of myb1 like 2 membrane trafficking protein, TOM1L2; kinesin family member 3A, KIF3A). Furthermore, cytochrome b (CYTB), dedicator of cytokinesis 6 (DOCK6), cyclin-dependent kinase 6 (CDK6), mitogen-activated protein kinase kinase 5 (MAP2K5), cilia and flagella associated protein 69 (CFAP69), myosin heavy chain 14 (MYH14), epithelial splicing regulatory protein 2 (ESRP2), activating signal cointegrator 1 complex subunit 3 (ASCC3), and remodeling and spacing factor 1 (RSF1), which may be involved in embryo and gamete development, embryo–maternal communication, and the transport process, are significantly enriched, and were highly expressed in the LH vs. the LL comparison group.

### 2.4. Parallel Reaction Monitoring (PRM) Analysis

To ensure the authenticity of the DIA LC-MS/MS strategy, we randomly selected six proteins from the oviduct proteome to perform parallel reaction monitoring (PRM) validation (Figure 4B), including drebrin 1 (DBN1), oviductin (OVN), PGAM family member 5, mitochondrial serine/threonine protein phosphatase (PGAM5), fetuin B (FETUB), SEC63 homolog, protein translocation regulator (SEC63), and progesterone receptor (PGR). Among them, fetuin B (FETUB), oviductin (OVN), and progesterone receptor (PGR) are related to fertilization, early embryonic development, and oviduct function [20,29,30] (Figure 4B, Supplementary Data S4). The PRM analysis revealed similarities between the trends of abundance between PRM and DIA, demonstrating the validity of the proteome data.

### 2.5. Bioinformatic Analysis of DAPs

The subcellular localization, domain enrichment, GO annotation, and Kyoto encyclopedia of genes and genomes (KEGG) enrichment analysis were used to understand the functions of the DAPs. We performed subcellular fractionation analyses, which revealed that 98 and 167 DAPs were classified into six subcellular localization categories in the FH vs. FL and LH vs. LL groups, respectively. In the FH vs. FL comparison groups, a total of 53 DAPs were localized to the nuclear (54.08%), followed by the cytoplasmic (30.61%) (Figure 4C). Regarding the LH vs. LL comparison, the subcellular localizations were like those in the FH vs. FL comparison of DAPs. Domain enrichment revealed that DAPs between FH and FL mainly included the LSM domain, protein phosphatase 2C, and some other domains (*p* < 0.01) (Figure 4D). Meanwhile, the most enrichened domains in the LH vs. LL were the PH domain, RhoGAP domain (*p* < 0.01), etc. (Figure 4E). It is important to emphasize that in sheep, changes in PH may be associated with periodic changes in mucin secretion in the oviduct [31].

**Figure 5:**
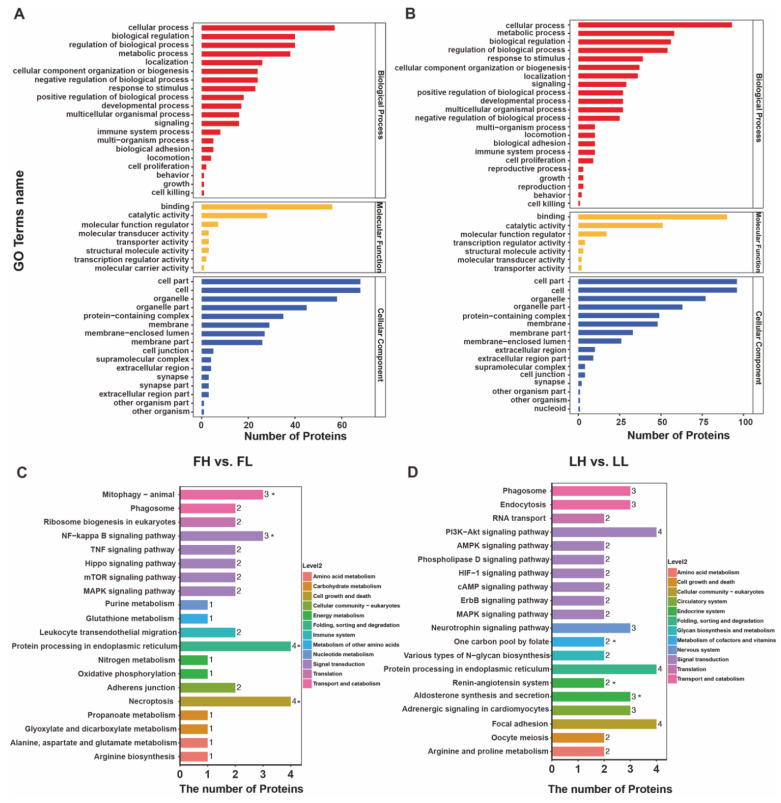
Gene ontology (GO) and Kyoto Encyclopedia of Genes and Genomes (KEGG) pathway enrichment analyses of differentially abundant proteins (DAPs) in the oviduct. GO annotation of DAPs in FH vs. FL (**A**) and LH vs. LL (**B**) comparison groups. KEGG classification of DAPs in FH vs. FL (**C**) and LH vs. LL (**D**) comparison groups. * Presents significant difference at *p* < 0.05.

GO enrichment analysis of all DAPs revealed that they were mostly localized in the cell part, and a large number of DAPs were mainly involved in molecular function with binding ability, catalytic activity, and molecular function regulation (Supplementary Data S3). Among the annotated terms of the FH vs. FL comparison group (Figure 5A), in the group within the GO BP category, a few DAPs were localized to biological adhesion, locomotion, cell proliferation, and growth, while many DAPs were assigned to respond to the negative regulation of biological processes. At the GO CC category level, 16 major processes were discovered from the DAPs, including the cell part, cell, and organelle. Binding, catalytic activity, and molecular function regulators were predominant in GO MF. Meanwhile, the main enriched GO categories in the case of LH vs. LL (Figure 5B) were essentially the same as in the FH vs. FL comparison groups. Noticeably, spindling 1 (SPIN1), cystathionine beta-synthase (CBS), and cilia and flagella-associated protein 69 (CFAP69) were enriched in the BP category of the reproductive process (GO:0022414) and reproduction (GO:0000003), similar to the LH vs. LL comparison groups.

As for the KEGG pathway analysis in the FH vs. FL, DAPs were enriched in protein processing in the endoplasmic reticulum, necroptosis, mitophagy–animal, and NF-kappa B signaling pathway (*p* < 0.01) (Figure 5C, Supplementary Data S3). In terms of LH vs. LL comparison, the pathways with the highest number of DAPs were the PI3K-Akt signaling pathway, protein processing in the endoplasmic reticulum, and focal adhesion (Supplementary Data S3). Furthermore, aldosterone synthesis and secretion, one carbon pool by folate, and the renin–angiotensin system were significantly enriched (*p* < 0.05) in KEGG terms belonging to the endocrine system (Figure 5D). Interestingly, the N-glycan biosynthesis pathway was also enriched in the tan module obtained by WGCNA analysis, reminding us that it is a specific function in the oviduct and needs further investigation. As noted above, the GO categories and KEGG pathways provided further clues to the molecular mechanisms of the oviduct underlying goat prolificacy.

### 2.6. Protein–Protein Interaction (PPI) Network Analysis for Goat Prolificacy Trait

**Figure 6:**
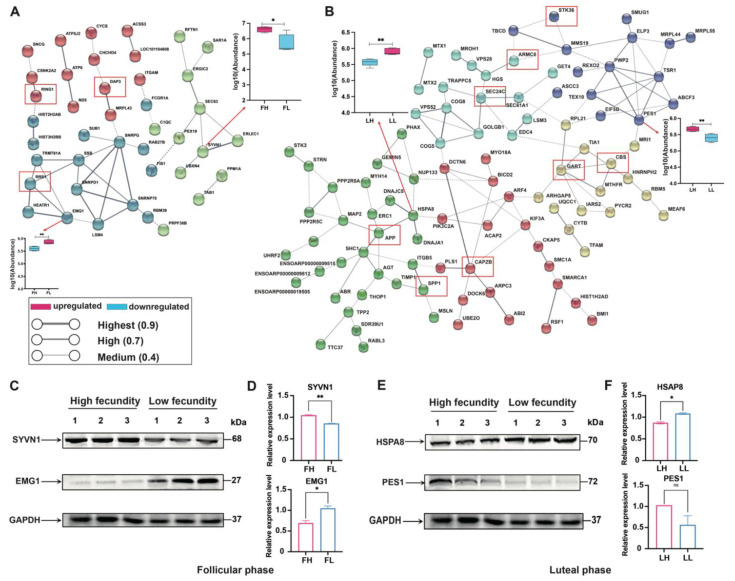
Protein–protein interactions (PPI) network and Western blot analyses of hub DAPs. (A and B) PPI analysis of the DAPs was conducted based on the STRING database (https://www.string-db.org/, accessed on 10 May 2022) in FH vs. FL (**A**) and LH vs. LL (**B**) comparison groups; we selected a confidence score of >0.4 to construct the PPI network; Nodes represent proteins, edges denote the predicted functional associations. Box plots of proteins SYVN1, EMG1, HSPA8, and PES1 are shown. Western blot analysis of SYVN1 and EMG1 in the follicular phase (**C**,**D**), and HSPA8 and PES1 in the luteal phase (**E**,**F**), and the reference protein GAPDH. * Presents significant difference at *p* < 0.05, ** presents significant difference at *p* < 0.01.

In the FH vs. FL comparison, an interaction network was constructed (Figure 6A, Supplementary Data S3), which can be divided into three clusters. It is shown that there were significantly enriched interactions among 41 DAPs, and SNRPG, SSB, SNRPD1, SNRNP70, TRMT61A, RRS1, SEC63, SYVN1, EMG1, RRS1, ERLEC1, ND5, DAP3, and RING1 as the hubs in the network. Meanwhile, in the LH vs. LL comparison, the interaction network was composed of 96 DAPs and divided into five clusters, among which PES1, GART, APP, TSR1, CBS, ELP3, SSP1, CAPZB, CYTB, CKAP5, ARMC8, SEC24C, RPL21, HSPA8, and STK36 were the center of the interaction network of each cluster (Figure 6B). In addition, trifunctional purine biosynthetic protein adenosine-3 (GART), heat shock protein family A (Hsp70) member 8 (HSPA8), F-actin-capping protein subunit beta (CAPZB), SEC24 homolog C, COPII coat complex component (SEC24C), and cystathionine beta-synthase (CBS) were also shown to be key proteins.

### 2.7. Western Blot Analysis of Hub Proteins

Given that differently characterized proteins were observed in the oviducts in the estrous cycles, we set out to identify specific protein expression signatures that could be used as future specific biomarkers affecting reproduction in the oviducts of goats. The expression of SYVN1, EMG1, HSPA8, and PES1 were subsequently validated in oviduct tissues by Western blotting and GAPDH as the reference protein. Coincidentally, the expression patterns of the four proteins in the Western blot experiments were highly consistent with the quantitative proteomics through the significant analysis (Figure 6C–F).

### 2.8. Combined Analysis of Proteome and Transcriptome (DAPs/DEGs) Responding to Prolificacy Trait of Yunshang Black Goat in Oviducts

**Figure 7:**
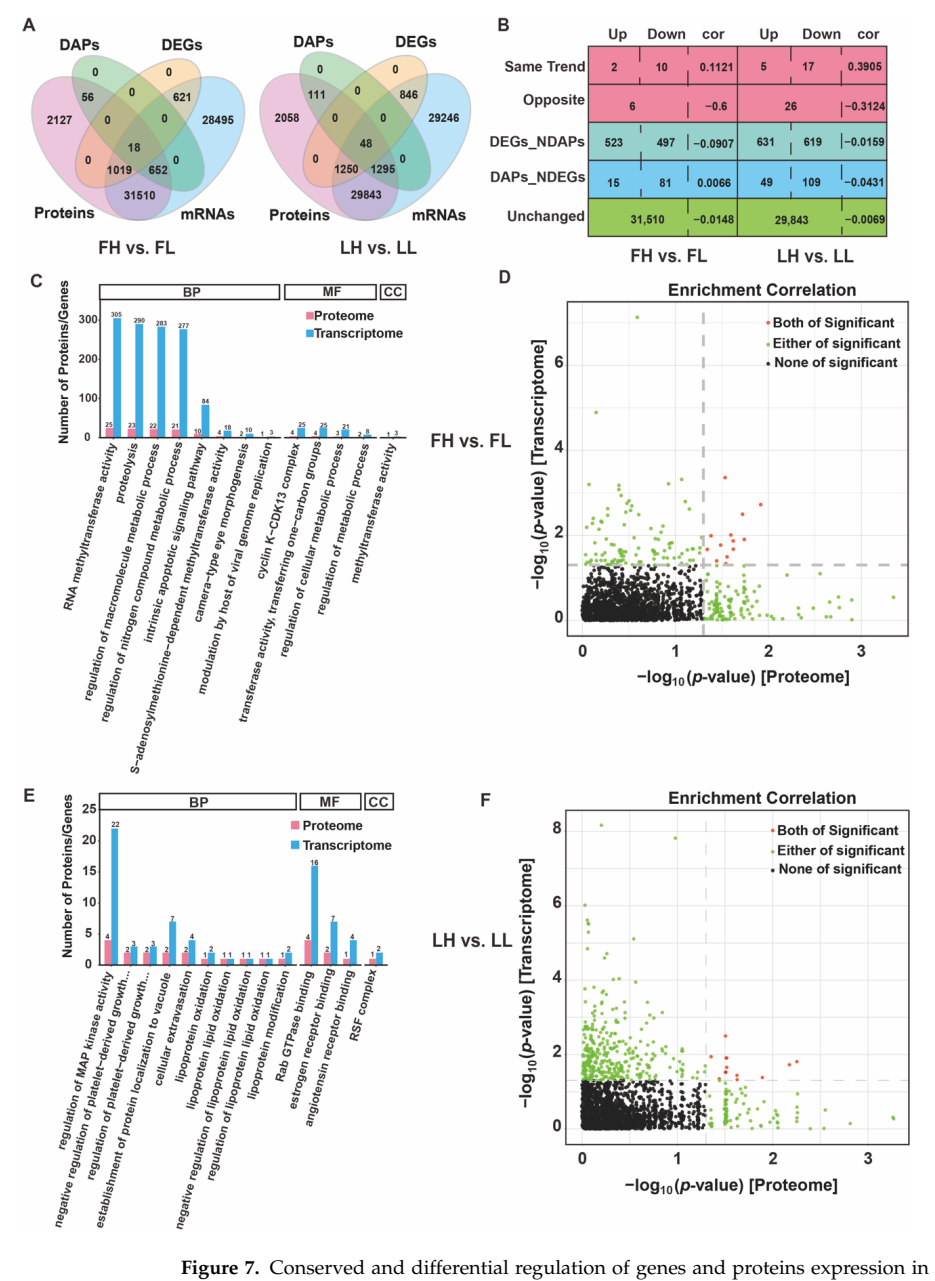
Conserved and differential regulation of genes and proteins expression in the oviduct. (**A**) Venn diagram regarding the proteins and genes that were abundant in the follicular and luteal phases. (**B**) Comparison of abundance ratios from transcriptomic and proteomic profiling in the follicular and luteal phases; cor represents Pearson correlation coefficient. GO enrichment analysis of DEGs correlated with proteomic data in the follicular (**C**) and luteal phase (**E**). GO terms enriched in the group of proteins that were significantly changed at both the mRNA and protein levels or proteins that were significantly changed at the mRNA or protein level in the follicular (**D**) and luteal phases (**F**).

Comparing the two phases, our transcriptomics and proteomics results showed that many more transcripts could be identified than proteins in the oviduct. In total, 62,322 and 62,591 genes (Supplementary Data S5) were detected from an RNA-seq analysis in goat oviduct in the follicular and luteal phases after stringent quality checks and data analysis, respectively, whereas the number of detected proteins was 4975 and 4851 (Supplementary Data S1). An integrated analysis of proteomic and transcriptomic profiling screened 33,199 and 32,436 genes overlapping in the mRNA and protein levels in the two phases, respectively (Figure 7A, Supplementary Data S5). Comparing the FH group with the FL group revealed 1658 differentially expressed genes (DEGs; 834 upregulated/824 downregulated), and 96 (number of correlation genes 726) DAPs were identified (Supplementary Data S1, S5). In LH vs. LL comparison groups, 2144 DEGs (1116 upregulated/1028 downregulated) and 167 (number of correlation genes 1455) DAPs (52 upregulated/115 downregulated) were identified (Supplementary Data S1, S5). The correlation analysis between transcripts and proteins showed that 33,199 and 32,436 protein–mRNA pairs were very poor and showed negative correlations (Figure S4, Supplementary Data S5). In FH vs. FL and LH vs. LL comparison groups, only 18 (12 are same trend with cor = 0.1121, and 6 are opposite with cor = −0.6) and 48 (22 are same trend, cor = 0.3905, and 26 are opposite, cor = −0.3124) pairs of DAPs-DEGs were in the oviduct, respectively (Figure 7B, Supplementary Data S5). In addition, 1020 and 1250 genes displayed altered expression in an mRNA-specific way in the FH vs. FL and LH vs. LL comparison groups, respectively, while a total of 96 and 158 proteins were differentially abundant at the protein level but not at the mRNA level. 

We focused GO analysis on the differentially expressed genes identified in the each proteomic and transcriptomic in the follicular and luteal phases, respectively (Figure 7C,E), of which were more significant at the mRNA level than the protein level (Figure 7D,F). These findings suggest that fecundity-related changes at the transcriptional level in the oviduct are more prevalent. As shown in Figure 7C,E, the DEGs/DEPs shared a common GO annotation pattern. For instance, proteolysis, intrinsic apoptotic signaling pathway, regulation of macromolecule metabolic process, regulation of the metabolic process, and regulation of cellular metabolic process were enriched in the follicular phase (Figure 7C). In the luteal phase, the combined enrichment of DEGs and DAPs was mainly shown in the regulation of MAP kinase activity, Rab GTPase binding, estrogen receptor binding, angiotensin receptor binding, and RSF complex (Figure 7E). These results suggest that many of the altered biological processes in the oviduct are driven, at least partially, by a combination of transcription and translation, and may be in response to goat fertility.

### 2.9. RT-qPCR Validation of Candidate DEGs

**Figure 8:**
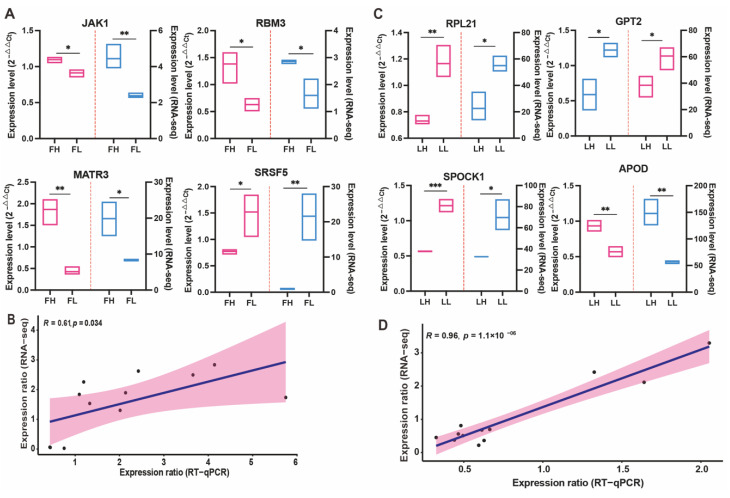
Protein and mRNA expression profiles of the protein candidates were validated by RT-qPCR in FH vs. FL (**A**) and LH vs. LL (**C**) comparison groups. Scatter plots show the correlation of gene expression between RNA-Seq and RT-qPCR in FH vs. FL (**B**) and LH vs. LL (**D**) comparison groups; Pearson correlation coefficient (R) and the *p* value is shown in the top left corner. * *p* < 0.05, ** *p* < 0.01, *** *p* < 0.001.

To further confirm the differences in gene expression at the transcriptome level, we selected 4 genes from the correlated DEGs in the FH vs. FL comparison to perform RT-qPCR validation with three replicates of each sample (Figure 8A), including *JAK1*, *RBM3*, *SRSF5*, and *MATR3*. Simultaneously, four genes were also detected in the LH vs. LL comparison group (Figure 8C), including *RPL21*, *GPT2*, *SPOCK1*, and *APOD*. The mean expression ratio was comparable between the RT-qPCR and RNA-Seq datasets. Linear regression analysis between RT-qPCR and RNA-seq showed an overall correlation coefficient (R) = 0.61 and 0.96 (Figure 8B,D) for FH vs. FL and LH vs. LL, respectively, which indicates the good correlation of these eight genes were consistent in transcriptomics databases.

## 3. Discussion

Mammalian reproduction is a complex process with coordinated regulation at multiple reproductive organs and molecular levels. In this study, mechanisms involved in the prolificacy trait of goats induced by oviduct with estrous periods were characterized using the DIA-based proteomic method and bioinformatic analyses. Results revealed that a deep proteome map of the oviduct in Yunshang black goats. We demonstrate that the known effects of oviducts, such as DAPs indicative of biological adhesion, gamete and embryo transport, and embryonic development, are present at the follicular or luteal phase. In addition, we also used a combined proteomic and transcriptomic profiling approach to identify coordinated-yet-specific changes in the oviduct during estrous periods, revealing the goat oviduct’s molecular mechanisms. Like previous studies that have been performed on other organisms [21,32,33,34], we utilized combinatorial functional mapping of goat oviducts.

Weighted gene co-expression network analysis (WGCNA) has the potential to reconstruct significant biological pathways from proteomics data by grouping all proteins into modules, regardless of fold-changes or statistical significance, while remaining independent of existing databases of functional annotation enrichment and protein–protein interactions [35]. The protein co-expression network shows that the module related to fecundity in the luteal phases is led by the hub proteins, including NUP107, ANXA11, MED20, COX2, AKAP13, and IFT140. Among these proteins, a functional study revealed that nucleoporin 107 (NUP107) is phosphorylated on its N-terminal sites in vivo during mitosis and can be efficiently dephosphorylated by trimeric protein phosphatase 2A-B55 upon mitotic exit, suggesting that NUP107 is involved in cell cycle progression regulation [36]. As a member of the annexins family, annexin A11 (ANXA11) belongs to Ca^2+^-regulated phospholipid-binding proteins widely expressed in eukaryotic cells, and are always implicated in mediating vesicle trafficking, cell division, differentiation, and cell growth [37,38,39]. Extracellular vesicles (EVs), biological nanoparticles that mediates cell communication and are present in oviductal fluid, play a crucial role in the communication between gamete–oviductal and embryo–oviductal cells [40]. The organs of the female reproductive system exhibit basal cyclooxygenase 2 (COX2) expression [41]. At both the mRNA and protein levels, COX2 expression positively correlated with estrogen receptor (ER) expression [42]. According to early reports, female mice lacking COX2 exhibit decreased ovulation and fertilized eggs [43]. Additionally, COX2 modulates the generation of reactive oxygen species [44] and inflammation and inhibits apoptosis [45]. A-kinase anchoring proteins (AKAPs) are a family of proteins with signaling platform functions; A-kinase anchoring protein 13 (AKAP13) is a member of the AKAP protein family that contains the C-terminal nuclear receptor interacting domain (NRID), which can bind estrogen receptors (ERs) and progesterone receptors (PRs) [46] and increase their ligand-dependent activity of those. Therefore, AKAP13 is usually considered one of the candidate genes for regulating female fertility. Intraflagellar transporter protein 140 (IFT140) is a member of the intraflagellar transport (IFT) protein family and is a conserved mechanism essential for the assembly and maintenance of most eukaryotic cilia and flagella. Ciliated and secretory cells are the two main cell types that constitute the oviductal epithelium [47], whereas embryo transport is realized by the complex interaction of smooth muscle contraction, cilia beat, and fluid flow in the oviduct [47,48]. Therefore, IFT140 might display a key role in maintaining the activity of the oviduct cilia. Interestingly, the significant enrichment in N-Glycan biosynthesis confirms the possibility of cell adhesion, migration, and other physiological processes [49], these are essential for maternal–embryo communication and the proper transport of embryos in the oviduct function [50]. Together, these results reflect the multifaceted control of prolificacy trait of Yunshang black goats’ oviducts, which are necessary to control the fertility of female mammals and meri further study.

In this study, we quantified 98 and 167 DAPs in the oviduct tissues derived from follicular and luteal phases of the estrus cycle, respectively, which represents a major contribution to the understanding of oviduct function in the *Capra hircus* species. For DAPs in the follicular and luteal phases, we focused on biological adhesion and transporter activity terms. The process of biological adhesion is indispensable for intercellular communication, signal transduction, proliferation, and apoptosis [51] that occur at follicular and luteal phases in the oviduct. As the site of the origin of mammalian life, the oviducts are involved in the first reproductive activities, many of which involve the formation of tight junctions between gametes, embryos, and reproductive organs highly dependent on the ability to adhere [50]. For example, oocytes form tight junctions with cumulus cells (CCs) in cumulus–oocyte complexes (COCs), allowing the flow of stimulating factors required for oocyte maturation [52]. After the sperm enters the oviduct, countercurrent movement occurs, and some sperm migrate directly to the ampulla and combine with the oocytes to complete fertilization, while most of them adhere to the oviductal epithelial cells (OECs), waiting for capacitation during ovulation [53]. At the same time, this process is crucial for successful fertilization, which can ensure that a few specific sperm reach the ampulla isthmus junction where fertilization takes place [54]. Obviously, this is not specific to one function of the oviduct at the follicular or luteal phase. The effect of the transport process on reproduction has been documented in the literature. Lopez-Ubeda et al. recently found that the main pathways affected by insemination were related to molecular transport, protein trafficking, and cell-to-cell signaling [55]. Thus, proteins involved in biological adhesion and transporter activity may play roles in reproduction and are associated with the kidding number of goats. 

During the follicular phase, compared with the FL group, proteins CAMSAP3, GLUL, SYVN1, ITGAM, PEX19, PSTPIP2, and ACSS3 were significantly upregulated in the FH group. At the same time, two of these proteins, CAMSAP3 [56] and ITGAM [57], participated in the ciliary transport function. As described above, the ciliary function is essential: through it, the oviduct completes gamete and embryo transport, and it is directly involved in reproductive regulation. Synoviolin 1 (SYVN1) was shown to promote the migration, invasion, and proliferation of liver cancer cells and always regulates endoplasmic reticulum stress, blood vessel growth, oxidative stress, and cell apoptosis [58], and therefore may also promote gamete maturation and migration, thereby negatively regulating goat fecundity. In addition, some reproduction-related proteins showed downregulation trends, such as EMG1, ND5, and RING1. Essential for mitotic growth 1 (EMG1) is a highly conserved nucleolar protein identified to be widely expressed during early mouse embryonic development, and the loss of EMG1 function in mice prevents embryonic development before the blastoderm stage [59]. ND5 is the mitochondrial-encoded respiratory chain I (NADH dehydrogenase) subunit 5, the deletion of which leads to respiratory chain disorders. Further, mitochondrial dysfunction may be responsible for female infertility because it affects not only sperm and oocyte quality but also the fertilization process and early embryonic development [60]. Ring finger protein 1 (RING1) has been suggested to play roles in regulating germ-cell-specific gene expression both in the male [61] and female germline [62], which occupy the central locations in the PPI network and act as hubs that interact with other DAPs.

Notably, oocytes enter the oviduct after ovulation, and in the luteal phase, changes in the expression of genes that play a role in cell growth and development occur in the oviduct, thus preparing for early embryo development and successful pregnancy [63]. Hydrogen sulfide (H_2_S) is a potent vasodilator and angiogenic factor with potent angiogenic activity, and cystathionine β-synthase (CBS) upregulation increases endogenous H_2_S production, which plays a role in uterine vasodilation and is involved in pregnancy [64,65]. The trophic role of the oviduct is widely supported during early mitotic active embryonic development [66], where the vasculature acts as a nutrient delivery system and therefore provides nutrients to the embryo and gametes in the oviduct. In mammalian cells, PES1 is associated with more than 50% of telomerase activity, and data suggest that PES1 is a key cofactor in telomerase assembly and can regulate telomerase activity and positively regulate cellular senescence and accretion [67]. In addition, we focused attention on the proteins in the luteal phase that were associated with embryonic development. ELP3 and SEC24C, two downregulated proteins have been previously described to have a direct embryo support function [68,69] and may be important mediators of maternal–embryonic communication for embryo development. Of note, among downregulated proteins, two proteins, SPP1 and HSPA8, have a known role in reproductive processes. Secreted phosphoprotein 1 (SPP1) usually binds to integrins to mediate cell–cell and cell–extracellular matrix communication and promote cell adhesion, migration, and differentiation; it is expressed in the mouse uterus and placenta, and it has been reported to provide nutrients for embryonic or fetal development by increasing angiogenesis within the decidua [70]. The conserved nature of heat shock 70 kDa protein 8 (HSPA8) between mammalian species suggests that this protein may represent a common biological mechanism for maintaining sperm survival in the oviduct [71]. These observations list the possible roles of the oviduct protein within the fecundity and merit further study.

In recent years, proteomics and transcriptomics methods have identified abundant proteins and genes, laying the groundwork for more precise and detailed descriptions of molecular processes and the elucidation of complex physiological processes and their genetic regulation [72,73]. Previous work has started uncovering the amounts of protein and mRNA expression in the same sample to better understand probable molecular mechanisms and identify reliable biomarkers of specific biological processes [24,74]. In this study, we utilized transcriptomic data generated in the oviduct from the same tissue sample, which we previously published [75,76], to complement the proteomic analysis. Comparing the two phases, our transcriptomics and proteomics results showed that many more transcripts can be identified than proteins in the oviduct. This situation is commonly reported, revealing the inconsistent or lagging relationship between transcription and translation levels [77]. In addition, a correlation analysis showed a poor negative correlation between the proteome and transcriptome, indicating an inconsistency between genes at the transcriptional level and protein abundance. The reason for the above can be explained by the fact that in the process of protein translation from mRNA, factors such as mRNA splicing, protein overturning, and protein degradation may lead to a poor correlation between mRNA expression patterns and their respective proteins [78]. Our results indicated that the mechanisms affecting fecundity in the luteal phase were more complex than in the follicular phase, as indicated by the differentially expressed genes and differentially abundant proteins in the oviduct. Meanwhile, we examined the functional annotations of the DEGs/DAPs, and the result indicated that some DEGs and DAPs shared a common GO annotation pattern. Metabolic pathways are extensively enriched during the follicular phase in preparation for the maintenance of a stable oviductal microenvironment and the reception of gametes. In the luteal phase, the regulation of MAP kinase activity, estrogen receptor binding, and angiotensin receptor binding were mainly enriched for DAPs and DEGs. MAP kinase is extensively involved in reproductive processes [79]; we suggested that the MAP kinase activity in the oviduct is one of the main reasons for the prolificacy trait of goats. Moreover, the estrogen receptor (ER) binding term might be one of the main functions of the oviduct during embryo transport [80]. The functional classification of the transcriptome and proteome can improve our understanding of the molecular physiology of the mammalian oviduct. 

## 4. Materials and Methods

### 4.1. Ethics Statement

In this research, animal samples were approved by the Institute of Animal Sciences, Chinese Academy of Agricultural Sciences, Beijing, China. Ethical oversight was given by the Animal Ethics Committee of the IAS-CAAS (No. IAS2019-63).

### 4.2. Animals and Sample Acquisition

We obtained tissue specimens of Yunshang black goats, known as a new breed of domestic goat in China with high fecundity trait, which were raised in Yixingheng Animal Husbandry Technology Co., Ltd. Tuanjie Township Base in Kunming City (Yunnan, China). Twenty healthy female Yunshang black goats (ages ranged between 2 and 3 years old) with an average weight of 52.22 ± 0.43 kg were selected and divided into high-fecundity group (H: n = 10, kidding number = 3.3 ± 0.11), and low-fecundity group (L: n = 10, kidding number = 1.75 ± 0.08), according to their kidding number records. All Yunshang black goats were treated with simultaneous estrus based on the progesterone vaginal suppository during the nonpregnant period. Briefly, a controlled internal drug release (CIDR, Inter Ag Co., Ltd. Hamilton, New Zealand) device (progesterone 300 mg) was inserted into the vagina for 16 days to synchronize estrus in April 2020, then the vaginal sponge was removed, and the removal time was set to 0 h. Then, ten goats (high fecundity, n = 5; FL: low fecundity, n = 5) were euthanized (intravenous pentobarbital 100 mg/kg) within 48 h and were considered to be in the follicular phase (FH and FL) and another ten goats (high fecundity, n = 5; low fecundity, n = 5) were euthanized (intravenous pentobarbital 100 mg/kg) within 168 h, which corresponds to the luteal phase (LH and LL). All fresh oviduct tissues were immediately collected in the follicular and luteal phases, respectively, and were washed by ice-cold phosphate-buffered saline (PBS) to remove contaminates, rapidly frozen with liquid nitrogen, and stored in a −80 °C refrigerator until protein extractions.

### 4.3. Protein Preparation and Fractionation for Data-Dependent Acquisition (DDA) Library Generation

Twenty oviduct tissues were first homogenized with a MP FastPrep-24 homogenizer (24 × 2, 6.0 M/S, 60 s, twice), and then SDT buffer (4% SDS, 100 mM DTT, 150 mM Tris-HCl pH 8.0) was added. Further, the lysates were sonicated and boiled for 15 min. After centrifuging at 14,000× *g* for 40 min, the supernatant was quantified with the BCA Protein Assay Kit (Bio-Rad, Hercules, CA, USA), according to the manufacturer’s protocol. Then, the protein samples were stored at −80 °C. An equal aliquot of the 20 samples in this experiment were pooled into one sample for generating a DDA spectral library and quality control. Protein digestion was performed according to the FASP procedure described by Wisniewski [81]. Briefly, an equal quantity of proteins in each sample was incorporated into SDT buffer (4% SDS, 100 mM DTT, 150 mM Tris-HCl pH 8.0). The detergent, DTT, and other low-molecular-weight components were removed using UA buffer (8 M Urea, 150 mM Tris-HCl pH 8.0) by repeated ultrafiltration (Microcon units, 10 kD). Then 100 μL iodoacetamide (100 mM IAA in UA buffer) was added to block reduced cysteine residues and the samples were incubated for 30 min in a dark environment. The filters were washed with 100 μL UA buffer three times and then 100 μL 25 mM NH_4_HCO_3_ buffer twice. Finally, the protein suspensions were digested with 4 μg trypsin (Promega, Madison, WI, USA) in 40 μL 25 mM NH_4_HCO_3_ buffer overnight at 4 °C, and the resulting peptides were collected as a filtrate. The peptides of each sample were desalted on C18 Cartridges (Empore^TM^ SPE Cartridges C18 (standard density), bed I.D. 7 mm, volume 3 mL, Sigma, St. Louis, MI, USA), concentrated by vacuum centrifugation and reconstituted in 40 µL of 0.1% (*v/v*) formic acid. The peptide content was estimated by UV light spectral density at 280 nm using an extinction coefficient of 1.1 of 0.1% (g/L) solution that was calculated based on the frequency of tryptophan and tyrosine in vertebrate proteins.

Digested pool peptides were then fractionated to 10 fractions using Thermo Scientific^TM^ Pierce^TM^ High pH Reversed-Phase Peptide Fractionation Kit [82]. Each fraction was concentrated by vacuum centrifugation and reconstituted in 15µL of 0.1% (*v/v*) formic acid. Collected peptides were desalted on C18 Cartridges (Empore^TM^ SPE Cartridges C18 (standard density), bed I.D. 7 mm, volume 3 mL, Sigma) and reconstituted in 40µL of 0.1% (*v/v*) formic acid. The iRT-Kits (Biognosys, Schlieren, Switzerland) were added to correct the relative retention time differences between runs with volume proportion 1:3 for iRT standard peptides versus sample peptides.

### 4.4. Data-Dependent Acquisition (DDA) Mass Spectrometry Assay

All fractions for DDA library generation were injected on a Thermo Scientific Q Exactive HF X mass spectrometer connected to an Easy nLC 1200 chromatography system (Thermo Scientific). The peptide was first loaded onto an EASY-Spray TM C18 Trap column (Thermo Scientific, P/N 164946, 3 μm, 75 μm × 2 cm), then separated on an EASY-SprayTM C18 LC Analytical Column (Thermo Scientific, ES802, 2 μm, 75 μm × 25 cm) with a linear gradient of buffer B (84% acetonitrile and 0.1% Formic acid) at a flow rate of 250 nl/min over 90 min. MS detection method was positive ion, the scan range was 300–1800 *m/z*, resolution for MS1 scan was 60,000 at 200 *m/z*, target of AGC (Automatic gain control) was 3 e6, maximum IT was 25 ms, dynamic exclusion was 30.0 s. Each full MS–SIM scan followed 20 ddMS2 scans. Resolution for MS2 scan was 15,000, AGC target was 5 e4, maximum IT was 25 ms and normalized collision energy was 30 eV.

### 4.5. Data-Independent Acquisition (DIA) Mass Spectrometry Assay

The peptides of each sample were analyzed by LC-MS/MS operating in the data-independent acquisition (DIA) mode by Shanghai Applied Protein Technology Co., Ltd. Each DIA cycle contained one full MS–SIM scan, and 30 DIA scans covered a mass range of 350–1800 *m/z* with the following settings: SIM full scan resolution was 120,000 at 200 *m/z*; AGC 3e6; maximum IT 50 ms; profile mode; DIA scans were set at a resolution of 15,000; AGC target 3e6; Max IT auto; normalized collision energy was 30 eV. Runtime was 90 min with a linear gradient of buffer B (80% acetonitrile and 0.1% Formic acid) at a flow rate of 250 nl/min. QC samples (pooled samples from equal aliquots of each sample in the experiment) were injected in the DIA mode at the beginning of the MS study and after every 6 injections throughout the experiment, which was used to monitor the MS performance. 

### 4.6. Raw Mass Spectrometry Data Analysis

For DDA library data, the FASTA sequence database was searched with Spectronaut software (Spectronaut Pulsar X^TM^_12.0.20491.4, Biognosys). The database was downloaded at website: http://www.uniprot.org.iRT, accessed on 20 October 2021. The following peptides sequence was also added (>Biognosys|iRT-Kit| Sequence_fusionLGGNEQVTRYILAGVENSKGTFIIDPGGVIRGTFIIDPAAVIRGAGSSEPVTGLDAKTPVISGGPYEYRVEATFGVDESNAKTPVITGAPYEYRDGLDAASYYAPVRADVTPADFSEWSKLFLQFGAQGSPFLK). The parameters were set as follows: enzyme was trypsin, max missed cleavages were 2, the fixed modification was carbamidomethyl (C), and the dynamic modification was oxidation (M) and acetyl (Protein N-term). All reported data were based on 99% confidence for protein identification as determined by the false discovery ratrunsee (FDR = N(decoy) × 2/(N(decoy) + N(target)) ≤ 1%. The spectral library was constructed by importing the original raw files and DDA search results into Spectronaut Pulsar X TM_12.0.20491.4 (Biognosys).

DIA data were analyzed with Spectronaut^TM^ 14.4.200727.47784, searching the above constructed spectral library. Main software parameters were set as follows: retention time prediction type was dynamic iRT, interference on MS2 level correction was enabled, and cross-run normalization was enabled. All results were filtered based on a Q value cutoff of 0.01 (equivalent to FDR < 1%). After normalized to total peak intensity, the processed data were uploaded before importing into SIMCA-P (version 14.1, Umetrics, Umea, Sweden), which was subjected to multivariate data analysis. We performed the Pareto-scaled principal component analysis (PCA) of quantified proteins to visualize the relationship between the oviduct samples obtained from different fecundity goats using R packages.

### 4.7. Weighted Gene Co-expression Network Analysis

We applied the weighted gene co-expression network analysis (WGCNA) method in R package (Version 1.69) [83] to identify distinct protein modules among all identified proteins, with the following parameters: corType = pearson; minModuleSize = 30; mergeCutHeight = 0.1. A weighted protein co-expression network was generated using the log_2_ protein abundance sample matrix. The R software package gplots was used to perform a hierarchical clustering analysis and identify the sample outliers. After performing the necessary soft threshold power calculations, the standard scale-free network was set up. The closer that correlation value was to 1 and the correlation test *p* value of <0.05, meaning the module was the important one in determining the trait, the more relevant the models or networks created using WGCNA were to the external sample features. Next, tissue-specific indicators were found using correlation functional networks. Proteins were grouped hierarchically to create a dynamic tree-cut technique to identify the modules. The protein information in each module was then extracted using WGCNA, which was utilized to build the protein co-expression modules. The sequences of the proteins were locally searched using the NCBI BLAST+ client software (NCBI-blast-2.2.28+-win32.exe) and InterProScan to find homolog sequences, then GO annotation, KEGG enrichment, and co-expressed protein network analyses were performed to analyze, identify, and interpret diverse biological functions and hub proteins in the functionally related modules. 

### 4.8. Data Analysis and Identification of Differentially Abundant Proteins 

Differential proteome analysis between high- and low-fecundity goats’ oviducts of each phase were conducted. Proteins with a fold change (FC) >1.5 or <0.67 and *p* value of <0.05 in comparable groups (FH vs. FL or LH vs. LL) were considered differentially abundant proteins (DAPs). Volcano plots were generated using –log_10_(*p* value) as the y-axis and ±log_2_(|FC|) as the x-axis. Cluster software (R version 3.6.3) and Java Treeview software were used to perform hierarchical clustering analysis. Euclidean distance algorithm for similarity measure and average linkage clustering algorithm (clustering uses the centroids of the observations) for clustering were selected when performing hierarchical clustering.

### 4.9. Functional Annotation of Differentially Abundant Proteins

The functions of differentially abundant proteins were annotated to further explore their impact on biological processes. CELLO (http://cello.life.nctu.edu.tw/, accessed on 11 April 2022), which is a multi-class SVM classification system, was used to predict protein subcellular localization. Protein sequences were searched using the InterProScan software [82] to identify protein domain signatures from the InterPro member database Pfam. Domain enrichment analysis of differentially expressed proteins was performed using Fisher (Fisher’s Exact Test), considering the whole quantified proteins as the background dataset. Benjamini–Hochberg correction for multiple testing was applied to adjust derived *p* values.

Gene ontology (GO) terms were mapped, and sequences were annotated using the software program Blast2GO [84] (https://www.blast2go.com/, accessed on 5 May 2022). The GO annotation results were plotted by R scripts. Next, following annotation steps, the studied proteins were blasted against the online Kyoto encyclopedia of genes and genomes (KEGG) database (http://geneontology.org/, accessed on 5 May 2022) to retrieve their KEGG identifications and were subsequently mapped to pathways in KEGG. The protein–protein interaction (PPI) information of the DAPs was retrieved from the STRING online tool (http://string-db.org/, accessed on 10 May 2022, confidence score ≥ 0.4 and classified through k-means). Furthermore, the degree of each protein was calculated to evaluate the importance of the protein in the PPI network.

### 4.10. Transcriptome Sequencing

High-throughput RNA sequencing (RNA-seq) was conducted following our previously published studies [75,76] and using the same oviduct samples as in protein extraction. Briefly, total RNAs from the 20 oviduct tissues in follicular and luteal phases were extracted by TRIzol reagent (Invitrogen, Carlsbad, CA, USA), respectively. The RNA concentration and purity were measured using a NanoDropTM 2000 (Thermo Scientific^TM^, Wilmington, DE, USA) instrument, and RNA integrity was evaluated using an Agilent 2100 System (Agilent Technologies, Santa Clara, CA, USA). After quality assessment, these RNA samples were sent to Wuhan Frasergen Bioinformatics Co., Ltd. (Wuhan, China) for cDNA library construction and then sequenced on the Illumina NovaSeq platform. Immediately, HISAT2 (v2.1.0) [85] was used to map the clean reads of each sample to the reference genome. Only the uniquely mapped reads were assembled, and the expression levels were predicted using String Tie software (v.1.3.5) [86]. Next, the FPKM (fragments per kilobase of exon model per million mapped fragments) for each gene was obtained. DESeq2 [87] was used to calculate fold change and *p* value based on the normalized counts. The threshold of adjusted *p* value < 0.05 and fold change >1.5 or <0.67 were used to screen significantly differentially expressed genes (DEGs).

### 4.11. Correlation Analyses of Transcriptome and Proteome Profiles

We performed an in-depth analysis of 20 oviduct tissues from goats to understand the oviduct function and facilitate the discovery of prolificacy-related biomarkers, and the sketch map of correlation analyses of the transcriptome and proteome is shown in Supplementary Figure S1. A joint analysis of the proteome and transcriptome will help us grasp gene expression regulation in general. If an mRNA and its corresponding protein were expressed at the same phase, they were considered correlated in the high- and low-fecundity comparison groups, and if the expression levels of both an mRNA and its corresponding protein are significantly different at the same phase, they were defined as differentially expressed associated transcripts (DEG; threshold of fold change >1.5 or <0.67 and *p* < 0.05) and proteins (DAP; threshold of fold change > 1.5 or <0.67 and *p* < 0.05), respectively. The expression matrix of all genes/proteins was converted into log_2_ format. Two omics databases for the comparison groups of FH vs. FL and LH vs. LL were calculated by Spearman correlation coefficients, respectively. In addition, if the expression levels of both a gene and its corresponding protein were significantly different at the same phase, they were defined as differentially expressed associated transcripts and proteins, respectively. GO annotation and KEGG pathway analysis were then conducted for DEGs and DAPs.

### 4.12. Validation of Proteomic Data with Parallel Reaction Monitoring (PRM)

Six proteins’ expression levels selected from DIA quantitative proteomics analysis were validated using LC-PRMMS at Shanghai Applied Protein Technology Co., Ltd. (Shanghai, China). Peptides were prepared according to the DIA protocol, and the internal standard reference was a Peptide Retention Time Calibration Mixture (thermo) stable isotope peptide spiked in each sample. Prior to reversed-phase chromatography on an Easy nLC-1200 system, tryptic peptides were loaded on C18 stage tips for desalting (Thermo Scientific). One-hour liquid chromatography gradients with acetonitrile ranging from 5 to 35% in 45 min were used. A PRM analysis was carried out using a Q Exactive HF mass spectrometer (Thermo Scientific). Experimentally, using unique peptides of high intensity and confidence for each target protein, methods optimized for collision energy, charge state, and retention times for the most significantly regulated peptides were generated. The mass spectrometer was set to positive ion mode with the following parameters: a resolution of 70,000 (at 200 *m/z*), automatic gain control (ACG) target values of 3.0 × 10^−6^, and a maximum ion injection time of 200 ms. Following full MS scans, 20 PRM scans were performed at 35,000 resolution (at *m/z* 200), with AGC 3.0 × 10^−6^ and a maximum injection time of 200 ms. A 1.5 Th window was used to isolate the targeted peptides. Ion activation/dissociation was carried out in a higher energy dissociation (HCD) collision cell at a normalized collision energy of 27. Skyline (MacCoss Lab, University of Washington) [88] was used to analyze the raw data, and signal intensities for individual peptide sequences for each of the significantly altered proteins were quantified relative to each sample and normalized to a standard reference.

### 4.13. Western Blot Analysis

The total protein of oviductal tissue was extracted using the proteinase inhibitor-containing lysis buffer. The 4% SurePAGE gel (GenScript, Nanjing, China) was used to separate the equivalent amounts of protein. The separated proteins were placed onto a PVDF membrane (Pall, Mexico) after electrophoresis, and then blocked with sealing solution (Tiangen, Beijing, China). The blocked membrane was incubated overnight at 4 °C. These primary antibodies were anti-synoviolin 1 (SYVN1, 1:2500; Proteintech, Chicago, IL, USA), anti-N1-specific pseudouridine methyltransferase (EMG1, 1:1500; Proteintech, anti-heat shock protein family A (Hsp70) member 8 (HSPA8, 1:4000; Proteintech), and anti-pescadillo ribosomal biogenesis factor 1 (PES1, 1:6000; Proteintech), anti-GAPDH, while horseradish peroxidase (HRP)-conjugated affinipure Goat anti-rabbit IgG (H+L, 1:2000, Proteintech) was used as the secondary antibodies. According to the manufacturer’s instructions, Western blots were visualized on the Odyssey CLX imaging system (Li-COR) (Bio-Rad, USA) with a Supersignal HRP chemiluminescent substrate (Beyotime, Jiangsu, China). The band densities were digitally measured using ImageJ software (NIH).

### 4.14. Measuring mRNA Levels Using Real-Time Quantitative PCR

Four co-expressed DE mRNAs were randomly screened from per group and quantified. The primers of mRNAs were designed by Primer Premier 6 software and RPL19 was used as the reference gene, primer information is shown in Table 1. All primers were synthesized by Sangon Biotech (Shanghai, China). Three replicates of each sample were analyzed, and standard curves were established using the Roche Light Cycler^®^ 480II system (Roche Applied Science, Mannheim, Germany). Relative expression level of mRNA was calculated using the comparative cycle threshold (2^–ΔΔCt^) method [89] dependent on the *t*-test. 

### 4.15. Statistical Analysis

The normality of experiment data was investigated with the Shapiro–Wilk normality test prior to all statistical analyses. According to the results of the normality investigation, the difference in the expression level in the FH vs. FL and LH vs. LL comparison groups was analyzed by a Student’s t-test. Statistical analyses were calculated using SPSS v.25.0, and GraphPad Prism v.9.3.1 was performed to visualize the data. Spearman rank correlation analysis was performed to assess the correlation between proteins and mRNAs’ abundance in the oviduct. Each experiment was performed with at least 3 independent biological replicates; all data were expressed as means ± standard error of the mean (SEM). *P* values of < 0.05 indicate a statistical significance and are presented as * *p* < 0.05, ** *p* < 0.01, and *** *p* < 0.001.

## 5. Conclusions

Our analysis provides comprehensive insight into the underlying mechanisms by which oviduct function causes differences in the fecundity of goats. The WGCNA revealed that the oviduct function during the luteal phase might play an important role in the prolificacy trait. Meanwhile, the stage-specific changes of proteins were identified, which expanded our understanding of the oviductal maternal–gamete communication factors in regulating kidding numbers. Additionally, we utilized the linkage between the proteome and transcriptome to suggest the main process for oviductal executive functions. The genes at both levels are closely associated with the same stage-specific functions, such as metabolic processes, MAP kinase activity, estrogen receptor binding, and angiotensin receptor binding, highlighting the importance of combining different gene-expression measurements. In future works, candidate biomarkers need to be added or overexpressed in the in vitro co-culture of oviductal cells and gametes/embryos to validate the function of these genes.

## Figures and Tables

**Table 1:** Primers were designed for expression analysis in this study.

Gene Name	Primer Sequence (5′-3′)	Product Size (bp)
JAK1	F: CGGAGAGTACACAGCGGAGGAG	124
	R: GCGATTCGGAGCATACCAGAGC	
HOOK2	F: GCCGACAAGCACTGATGGATCTC	115
	R: GGCAGAGACTGTGACTTCAAGGTTC	
SRSF5	F: GCTAAGTGCGTCAGTTGTGGAGAG	90
	R: GAGAGGTCCGTAGTCTGCGATAGAG	
MATR3	F: GCAGTCTACAAACCCAGCACCAG	107
	R: TTCCATTTCCAGCACCCAGATTTCC	
RPL21	F: GAGCCGAGATAGCTTCCTGAAACG	145
	R: GGGTTCCTTTCCATTGGTCCTCAC	
GPT2	F: GCCAGCAGCCAATCACCTTCC	131
	R: GTTCCCACCGCAAGCCTGTAG	
SPOCK1	F: AGTGACGGAGGATGACGAGGATG	132
	R: AGACAAATGCAGGAACGGGAAGTG	
APOD	F: AGAAGGGAAATGTGGCAGCAGAAG	98
	R: TCAGACACGCAAGGTAACAGAATGG	
RPL19	F: ATCGCCAATGCCAACTC	154
	R: CCTTTCGCTTACCTATACC	

## Data Availability

The mass spectrometry data of proteomics and PRM have been deposited to the ProteomeXchange Consortium (http://proteomecentral.proteomexchange.org, accessed on 14 November 2022) via the iProX partner repository with the dataset identifier PXD038122. The RNA-seq raw data generated in this study are available from the NCBI Sequence Read Archive (SRA; http://www.ncbi.nlm.nih.gov/sra, accessed on 14 July 2022) under accession number: PRJNA854769.

## Supplementary Materials

The following supporting information can be downloaded at: https://www.mdpi.com/article/10.3390/ijms232314888/s1, Figure S1: Experimental design and work flow for the integrative proteomics and transcriptomics comparison of high- and low-fecundity goat oviducts from Yunushang black goat in the follicular and luteal phases; Figure S2: Quality control (QC) and quantitative heat map of DIA; Figure S3: Weighted correlation network analysis (WGCNA); Figure S4: Combined Analysis of Proteome and Transcriptome; Table S1: Peptide quality statistics; Table DataS1: The identification and quantitative results of DIA; Table DataS2: WGCNA analysis; Table DataS3: Bioinformatics analysis; Table DataS4: Parallel Reaction Monitoring (PRM) and RT-qPCR Validation results; Table DataS5: Proteome and transcriptome association analysis.

## References

[1] Shah A.M., Cai Y.M., Zou H.W., Zhang X.F., Wang L.Z., Xue B., Yu P.Q., Wang Z.S., Peng Q.H. (2019). Effects of supplementation of branches and leaves trimmed from tea plant on growth performance, rumen fermentation and meat composition of Nanjiang yellow goats. Animals.

[2] Bridi A., Perecin F., da Silveira J.C. (2022). Extracellular vesicles mediated early embryo–maternal interactions. Int. J. Mol. Sci..

[3] Fazeli A. (2008). Maternal communication with gametes and embryos. Theriogenology.

[4] Li S., Winuthayanon W. (2017). Oviduct: Roles in fertilization and early embryo development. J. Endocrinol..

[5] Gao Y.W., Liu X.Y., Tang B., Li C., Kou Z.H., Li L., Liu W.Q., Wu Y., Kou X.C., Li J.Y. (2017). Protein expression landscape of mouse embryos during pre-implantation development. Cell. Rep..

[6] Stitzel M.L., Seydoux G. (2007). Regulation of the oocyte-to-zygote transition. Science..

[7] Besenfelder U., Havlicek V., Brem G. (2012). Role of the oviduct in early embryo development. Reprod. Domest. Anim..

[8] Kenngott R.A., Sinowatz F. (2007). Prenatal development of the bovine oviduct. Anat. Histol. Embryol..

[9] Goudet G. (2011). Fertilisation in the horse and paracrine signalling in the oviduct. Reprod. Fertil. Dev..

[10] Lee S.H., Song E.J., Hwangbo Y., Lee S., Park C.K. (2016). Change of uterine histroph proteins during follicular and luteal phase in pigs. Anim. Reprod. Sci..

[11] Coy P., García-Vázquez F.A., Visconti P.E., Avilés M. (2012). Roles of the oviduct in mammalian fertilization. Reproduction.

[12] Mondejar I., Acuna O.S., Izquierdo-Rico M.J., Coy P., Aviles M. (2012). The oviduct: Functional genomic and proteomic approach. Reprod. Domest. Anim..

[13] Seytanoglu A., Georgiou A.S., Sostaric E., Watson P.F., Holt W.V., Fazeli A. (2008). Oviductal cell proteome alterations during the reproductive cycle in pigs. J. Proteome Res..

[14] Hu J.J., Xiao L.F., Song L.L., Ge W.B., Duan H.W., Jiang Y. (2020). The expression of melatonin receptors MT1 and MT2 is regulated by E2 in sheep oviduct. Gen. Comp. Endocrinol..

[15] Lv J.S., Ge W.B., Ding Z.Q., Zeng J.L., Wang W.J., Duan H.W., Hu J.J., Zhang Y., Zhao X.X. (2022). Expression of dihydrotestosterone synthases and androgen receptor in sheep oviduct ampulla and its regulation by estradiol and progesterone. Reprod. Biol..

[16] Lapointe J., Kimmins S., Maclaren L.A., Bilodeau J.F. (2005). Estrogen selectively up-regulates the phospholipid hydroperoxide glutathione peroxidase in the oviducts. Endocrinology.

[17] Binelli M., Gonella-Diaza A.M., Mesquita F.S., Membrive C.M.B. (2018). Sex steroid-mediated control of oviductal function in Cattle. Biology.

[18] McNutt T.L., Olds-Clarke P., Way A.L., Suarez S.S., Killian G.J. (1994). Effect of follicular or oviductal fluids on movement characteristics of bovine sperm during capacitation in vitro. J. Androl..

[19] Satake N., Elliott R.M., Watson P.F., Holt W.V. (2006). Sperm selection and competition in pigs may be mediated by the differential motility activation and suppression of sperm subpopulations within the oviduct. J. Exp. Biol..

[20] Pradeep M.A., Jagadeesh J., De A.K., Kaushik J.K., Malakar D., Kumar S., Dang A.K., Das S.K., Mohanty A.K. (2011). Purification, sequence characterization and effect of goat oviduct-specific glycoprotein on in vitro embryo development. Theriogenology.

[21] Abdulghani M., Song G.Y., Kaur H., Walley J.W., Tuteja G. (2019). Comparative analysis of the transcriptome and proteome during mouse placental development. J. Proteome Res..

[22] Schwanhausser B., Busse D., Li N., Dittmar G., Schuchhardt J., Wolf J., Chen W., Selbach M. (2011). Global quantification of mammalian gene expression control. Nature.

[23] Walley J.W., Sartor R.C., Shen Z.X., Schmitz R.J., Wu K.J., Urich M.A., Nery J.R., Smith L.G., Schnable J.C., Ecker J.R. (2016). Integration of Omic Networks in a Developmental Atlas of Maize. Science.

[24] La Y.F., Tang J.S., Guo X.F., Zhang L.P., Gan S.Q., Zhang X.S., Zhang J.L., Hu W.P., Chu M.X. (2020). Proteomic analysis of sheep uterus reveals its role in prolificacy. J. Proteom..

[25] Tang W., Zhou M., Dorsey T.H., Prieto D.A., Wang X.W., Ruppin E., Veenstra T.D., Ambs S. (2018). Integrated proteotranscriptomics of breast cancer reveals globally increased protein-mRNA concordance associated with subtypes and survival. Genome. Med..

[26] Zhang X.L., Xia M.L., Su X.J., Yuan P., Li X.K., Zhou C.Y., Wan Z.P., Zou W. (2021). Photolytic degradation elevated the toxicity of polylactic acid microplastics to developing zebrafish by triggering mitochondrial dysfunction and apoptosis. J. Hazard. Mater..

[27] Liao Q., Tang J.Q., Wang H.Y., Yang W.C., He L.X., Wang Y.Y., Yang Z.H. (2020). Dynamic proteome responses to sequential reduction of Cr(VI) and adsorption of Pb(II) by Pannonibacter phragmitetus BB. J. Hazard. Mater..

[28] Sun Z.P., Hong Q.H., Liu Y.F., He X.Y., Di R., Wang X.Y., Ren C.H., Zhang Z.J., Chu M.X. (2022). Characterization of circular RNA profiles of oviduct reveal the potential mechanism in prolificacy trait of goat in the estrus cycle. Front. Physiol..

[29] Fang L., Hu X.R., Cui L., Lv P.P., Ma X.Q., Ye Y.H. (2019). Serum and follicular fluid fetuin-B levels are correlated with fertilization rates in conventional IVF cycles. J. Assist. Reprod. Genet..

[30] Bylander A., Lind K., Goksor M., Billig H., Larsson D. (2013). The classical progesterone receptor mediates the rapid reduction of fallopian tube ciliary beat frequency by progesterone. Reprod. Biol. Endocrinol..

[31] Hadek R. (1953). Alteration of pH in the sheep’s oviduct. Nature.

[32] Wang H.D., Qi X.Y., Chen S.S., Feng J., Chen H.J., Qin Z.Y., Deng Y.M. (2021). An integrated transcriptomic and proteomic approach to dynamically study the mechanism of pollen-pistil interactions during jasmine crossing. J. Proteom..

[33] Wang C.P., Liu N.X., Geng Z., Ji M.J., Wang S.M., Zhuang Y.M., Wang D., He G., Zhao S.T., Zhou G.K. (2022). Integrated transcriptome and proteome analysis reveals brassinosteroid-mediated regulation of cambium initiation and patterning in woody stem. Hortic. Res..

[34] Xiao H.Y., Li G.C., Wang Z.Q., Guo Y.R., Liu N.Y. (2021). Combined transcriptomic, proteomic and genomic analysis identifies reproductive-related proteins and potential modulators of female behaviors in *Spodoptera litura*. Genomics.

[35] Carr A.V., Frey B.L., Scalf M., Cesnik A.J., Rolfs Z., Pike K.A., Yang B., Keller M.P., Jarrard D.F., Shortreed M.R. (2022). MetaNetwork enhances biological insights from quantitative proteomics differences by combining clustering and enrichment analyses. J. Proteome Res..

[36] Liu X.C., Han M.X., Xu Y., Wang H.Y., Li B. (2019). Knockdown of the premature ovarian insufficiency candidate gene NUP107 in ovarian granulosa cells affects cell functions, including receptor expression and estrogen synthesis. Reprod. Dev. Med..

[37] Smith B.N., Topp S.D., Fallini C., Shibata H., Chen H.J., Troakes C., King A., Ticozzi N., Kenna K.P., Soragia-Gkazi A. (2017). Mutations in the vesicular trafficking protein Annexin A11 are associated with amyotrophic lateral sclerosis. Sci. Transl. Med..

[38] Liao Y.C., Fernandopulle M.S., Wang G.Z., Choi H., Hao L., Drerup C.M., Patel R., Qamar S., Nixon-Abell J., Shen Y. (2019). RNA granules hitchhike on lysosomes for long-distance transport, using Annexin A11 as a molecular tether. Cell.

[39] Wang J.S., Guo C.M., Liu S.Q., Qi H.B., Yin Y., Liang R., Sun M.Z., Greenaway F.T. (2014). Annexin A11 in disease. Clin. Chim. Acta.

[40] Bridi A., Perecin F., da Silveira J.C. (2022). The role of the oviduct and extracellular vesicles during early embryo development in bovine. Anim. Reprod..

[41] Lyndin M., Kravtsova O., Sikora K., Lyndina Y., Kuzenko Y., Awuah W.A., Abdul-Rahman T., Hyriavenko N., Sikora V., Romaniuk A. (2022). COX2 Effects on endometrial carcinomas progression. Pathol. Res. Pract..

[42] Jarzabek K., Koda M., Walentowicz-Sadlecka M., Grabiec M., Laudanski P., Wolczynski S. (2013). Altered expression of ERs, aromatase, and COX2 connected to estrogen action in type 1 endometrial cancer biology. Tumour. Biol..

[43] Cheng J.G., Stewart C.L. (2003). Loss of cyclooxygenase-2 retards decidual growth but does not inhibit embryo implantation or development to term. Biol. Reprod..

[44] Xu Y., Yang X.Y., Wang T., Yang L., He Y.Y., Miskimins K., Qian S.Y. (2018). Knockdown delta-5-desaturase in breast cancer cells that overexpress COX-2 results in inhibition of growth, migration and invasion via a dihomo-γ-linolenic acid peroxidation dependent mechanism. BMC Cancer.

[45] Hosseini F., Mahdian-Shakib A., Jadidi-Niaragh F., Enderami S.E., Mohammadi H., Hemmatzadeh M., Mohammed H.A., Anissian A., Kokhaei P., Mirshafiey A. (2018). Anti-inflammatory and anti-tumor effects of α-l-guluronic acid (G2013) on cancer-related inflammation in a murine breast cancer model. Biomed. Pharm..

[46] Zhang X.W., Yuan R.R., Bai Y.Y., Yang Y.T., Song X.Y., Lan X.Y., Pan C.Y. (2021). A deletion mutation within the goat AKAP13 gene is significantly associated with litter size. Anim. Biotechnol..

[47] Jiang R.W., Tang X.F., Pan J.L., Li G.Z., Yang N.J., Tang Y.D., Bi S.L., Cai H., Chen Q.H., Chen D.J. (2022). CDC42 governs normal oviduct multiciliogenesis through activating AKT to ensure timely embryo transport. Cell. Death Dis..

[48] Ward S.M., Hwang S.J., Yan W., Offermanns S., Sanders K.M. (2022). Intrinsic pacemaker activity and propulsive forces provided by the myosalpinx are necessary for egg and embryo transport in the oviduct. Biol. Reprod..

[49] Nita-Lazar M., Noonan V., Rebustini I., Walker J., Menko A.S., Kukuruzinska M.A. (2009). Overexpression of DPAGT1 leads to aberrant N-glycosylation of E-cadherin and cellular discohesion in oral cancer. Cancer. Res..

[50] Budna-Tukan J., Swiatly-Blaszkiewicz A., Celichowski P., Kaluzna S., Konwerska A., Sujka-Kordowska P., Jankowski M., Kulus M., Jeseta M., Piotrowska-Kempisty H. (2019). “Biological Adhesion” is a significantly regulated molecular process during long-term primary in vitro culture of oviductal epithelial cells (Oecs): A transcriptomic and proteomic study. Int. J. Mol. Sci..

[51] Cal S., Freije J.M., Lopez J.M., Takada Y., Lopez-Otín C. (2000). ADAM 23/MDC3, a human disintegrin that promotes cell adhesion via interaction with the alphavbeta3 integrin through an RGD-independent mechanism. Mol. Biol. Cell..

[52] Kempisty B., Ziolkowska A., Ciesiolka S., Piotrowska H., Antosik P., Bukowska D., Nowicki M., Brussow K.P., Zabel M. (2014). Study on connexin gene and protein expression and cellular distribution in relation to real-time proliferation of porcine granulosa cells. J. Biol. Regul. Homeost. Agents.

[53] Mahe C., Zlotkowska A.M., Reynaud K., Tsikis G., Mermillod P., Druart X., Schoen J., Saint-Dizier M. (2021). Sperm migration, selection, survival, and fertilizing ability in the mammalian oviduct. Biol. Reprod..

[54] Tokuhiro K., Ikawa M., Benham A.M., Okabe M. (2012). Protein disulfide isomerase homolog PDILT is required for quality control of sperm membrane protein ADAM3 and male fertility [corrected]. Proc. Natl. Acad. Sci. USA.

[55] Lopez-Ubeda R., Garcia-Vazquez F.A., Romar R., Gadea J., Munoz M., Hunter R.H., Coy P. (2015). Oviductal transcriptome is modified after insemination during spontaneous ovulation in the sow. PLoS ONE.

[56] Usami F.M., Arata M., Shi D., Oka S., Higuchi Y., Tissir F., Takeichi M., Fujimori T. (2021). Intercellular and intracellular cilia orientation is coordinated by CELSR1 and CAMSAP3 in oviduct multi-ciliated cells. J. Cell Sci..

[57] Awamleh Z., Han V.K.M. (2020). Identification of miR-210-5p in human placentae from pregnancies complicated by preeclampsia and intrauterine growth restriction, and its potential role in the pregnancy complications. Pregnancy. Hypertens..

[58] Zhou W.Y., Yang F.S. (2022). Circular RNA circRNA-0039459 promotes the migration, invasion, and proliferation of liver cancer cells through the adsorption of miR-432. Bioengineered.

[59] Wu X.L., Sandhu S., Patel N., Triggs-Raine B., Ding H. (2010). EMG1 is essential for mouse pre-implantation embryo development. BMC Dev. Biol..

[60] Tatone C., Di Emidio G. (2022). Mitochondria biology in reproductive function. Antioxidants.

[61] Maezawa S., Hasegawa K., Yukawa M., Sakashita A., Alavattam K.G., Andreassen P.R., Vidal M., Koseki H., Barski A., Namekawa S.H. (2017). Polycomb directs timely activation of germline genes in spermatogenesis. Genes Dev..

[62] Yokobayashi S., Liang C.Y., Kohler H., Nestorov P., Liu Z., Vidal M., van Lohuizen M., Roloff T.C., Peters A.H. (2013). PRC1 coordinates timing of sexual differentiation of female primordial germ cells. Nature.

[63] Kim J.M., Park J.E., Yoo I., Han J., Kim N., Lim W.J., Cho E.S., Choi B., Choi S., Kim T.H. (2018). Integrated transcriptomes throughout swine oestrous cycle reveal dynamic changes in reproductive tissues interacting networks. Sci. Rep..

[64] Sheibani L., Lechuga T.J., Zhang H., Hameed A., Wing D.A., Kumar S., Rosenfeld C.R., Chen D.B. (2017). Augmented H2S production via cystathionine-beta-synthase upregulation plays a role in pregnancy-associated uterine vasodilation. Biol. Reprod..

[65] Qi Q.R., Lechuga T.J., Patel B., Nguyen N.A., Yang Y.H., Li Y., Sarnthiyakul S., Zhang Q.W., Bai J., Makhoul J. (2020). Enhanced stromal cell CBS-H2S production promotes estrogen-stimulated human endometrial angiogenesis. Endocrinology.

[66] Fujii D.T., Yohannes E., Por E.D., Gillette L., Beesley R.D., Heitmann R.J., Chow G.E., Burney R.O. (2021). The proteome of human Fallopian tube lavages during the phase of embryo transit reveals candidate proteins for the optimization of preimplantation embryo culture. Hum. Reprod..

[67] Cheng L., Yuan B., Ying S.Y., Niu C., Mai H., Guan X.X., Yang X.H., Teng Y., Lin J., Huang J.J. (2019). PES1 is a critical component of telomerase assembly and regulates cellular senescence. Sci. Adv..

[68] Yoo H., Son D., Jang Y.J., Hong K. (2016). Indispensable role for mouse ELP3 in embryonic stem cell maintenance and early development. Biochem. Biophys. Res. Commun..

[69] Adams E.J., Khoriaty R., Kiseleva A., Cleuren A.C.A., Tomberg K., van der Ent M.A., Gergics P., Tang V.T., Zhu G.J., Hoenerhoff M.J. (2021). Murine SEC24D can substitute functionally for SEC24C during embryonic development. Sci. Rep..

[70] Kramer A.C., Erikson D.W., McLendon B.A., Seo H., Hayashi K., Spencer T.E., Bazer F.W., Burghardt R.C., Johnson G.A. (2021). SPP1 expression in the mouse uterus and placenta: Implications for implantation. Biol. Reprod..

[71] Elliott R.M., Lloyd R.E., Fazeli A., Sostaric E., Georgiou A.S., Satake N., Watson P.F., Holt W.V. (2009). Effects of HSPA8, an evolutionarily conserved oviductal protein, on boar and bull spermatozoa. Reproduction.

[72] Wang Z.Y., Leushkin E., Liechti A., Ovchinnikova S., Mossinger K., Bruning T., Rummel C., Grutzner F., Cardoso-Moreira M., Janich P. (2020). Transcriptome and translatome co-evolution in mammals. Nature.

[73] Jiang L.H., Wang M., Li S., Jian R.Q., Xiao Li X., Chan J., Dong G.L., Fang H.Y., Robinson A.E., Consortium G. (2020). A quantitative proteome map of the human body. Cell.

[74] Zhao L.M., Li F.D., Zhang X.X., Zhang D.Y., Li X.L., Zhang Y.K., Zhao Y., Song Q.Z., Huang K., Xu D. (2022). Integrative analysis of transcriptomics and proteomics of longissimus thoracis of the Hu sheep compared with the Dorper sheep. Meat. Sci..

[75] Sun Z.P., Zhang Z.J., Liu Y.F., Ren C.H., He X.Y., Jiang Y.T., Ouyang Y.N., Hong Q.H., Chu M.X. (2022). Integrated analysis of mRNAs and long non-coding RNAs expression of oviduct that provides novel insights into the prolificacy mechanism of goat (*Capra. hircus*). Genes.

[76] Sun Z.P., Hong Q.H., Liu Y.F., Ren C.H., He X.Y., Jiang Y.T., Ouyang Y.N., Chu M.X., Zhang Z.J. (2022). Oviduct transcriptomic reveals the regulation of mRNAs and lncRNAs related to goat prolificacy in the luteal phase. Animals.

[77] Garg R., Shankar R., Thakkar B., Kudapa H., Krishnamurthy L., Mantri N., Varshney R.K., Bhatia S., Jain M. (2016). Transcriptome analyses reveal genotype-and developmental stage-specific molecular responses to drought and salinity stresses in chickpea. Sci. Rep..

[78] Zhang H.Y., Lei G., Zhou H.W., He C., Liao J.L., Huang Y.J. (2017). Quantitative iTRAQ-based proteomic analysis of rice grains to assess high night temperature stress. Proteomics.

[79] Luyao Zhang L.Y., Liu K.X., Qingrui Zhuan Q.R., Liu Z.Q., Lin Meng L., Fu X.W., Jia G.X., Hou Y.P. (2022). Mitochondrial calcium disorder affects early embryonic development in mice through regulating the ERK/MAPK pathway. Oxid. Med. Cell. Longev..

[80] Li S., O’Neill S.R.S., Zhang Y., Holtzman M.J., Takemaru K.I., Korach K.S., Winuthayanon W. (2017). Estrogen receptor α is required for oviductal transport of embryos. FASEB J..

[81] Wisniewski J.R., Zougman A., Nagaraj N., Mann M. (2009). Universal sample preparation method for proteome analysis. Nat. Methods.

[82] Rosenberger G., Koh C.C., Guo T., Rost H.L., Kouvonen P., Collins B.C., Heusel M., Liu Y.S., Caron E., Vichalkovski A. (2014). A repository of assays to quantify 10,000 human proteins by SWATH-MS. Sci. Data.

[83] Langfelder P., Horvath S. (2008). WGCNA: An R package for weighted correlation network analysis. BMC Bioinform..

[84] Shao Y.L., Zhou H.Z., Wu Y.R., Zhang H., Lin J., Jiang X.Y., He Q.J., Zhu J.S., Li Y., Yu H. (2019). OsSPL3, an SBP-Domain protein, regulates crown root development in rice. Plant Cell..

[85] Kim D., Langmead B., Salzberg S.L. (2015). HISAT: A fast spliced aligner with low memory requirements. Nat. Methods.

[86] Kovaka S., Zimin A.V., Pertea G.M., Razaghi R., Salzberg S.L., Pertea M. (2019). Transcriptome assembly from long-read RNA-seq alignments with StringTie2. Genome Biol..

[87] Love M.I., Huber W., Anders S. (2014). Moderated estimation of fold change and dispersion for RNA-seq data with DESeq2. Genome Biol..

[88] MacLean B., Tomazela D.M., Shulman N., Chambers M., Finney G.L., Frewen B., Kern R., Tabb D.L., Liebler D.C., MacCoss M.J. (2010). Skyline: An open source document editor for creating and analyzing targeted proteomics experiments. Bioinformatics.

[89] Livak K.J., Schmittgen T.D. (2001). Analysis of relative gene expression data using real-time quantitative PCR and the 2(-Delta Delta C(T)) Method. Methods.

