# Supplementary material for: Integrative Proteomics and Transcriptomics Profiles of the Oviduct Reveal the Prolificacy-Related Candidate Biomarkers of Goats (Capra hircus) in Estrous Periods
Source: Int J Mol Sci. 2022 Nov 28;23(23):14888. doi: 10.3390/ijms232314888 (PMC9737051; doi:10.3390/ijms232314888)
Supplement: Supplementary file 1 [file ijms-23-14888-s001.zip › SUPPORTING FIGURES.pdf]

SUPPORTING INFORMATION FOR

Integrative Proteomics and Transcriptomics Profiles of Oviduct Reveals the Prolificacy-Related Candidate Biomarkers of Goat in Different Estrus Periods

Zhipeng Sun[1,2], Yufang Liu[1], Xiaoyun He[1], Ran Di[1], Xiangyu Wang[1], Chunhuan Ren[1], Zijun Zhang[2,*], Mingxing Chu[1,*]

[1] Key Laboratory of Animal Genetics, Breeding and Reproduction of Ministry of Agriculture and Rural Affairs, Institute of Animal Science, Chinese Academy of Agricultural Sciences, Beijing 100193, China
[2] College of Animal Science and Technology, Anhui Agricultural University, Hefei 230036, China

*Corresponding authors
Mingxing Chu, Key Laboratory of Animal Genetics, Breeding and Reproduction of Ministry of Agriculture and Rural Affairs, Institute of Animal Science, Chinese Academy of Agricultural Sciences, No. 2 Yuanmingyuan West Rd., Beijing 100193, China.
Tel.: +86 10 62819850
Fax: +86 10 62895351
E-mail: mxchu@263.net

Zijun Zhang, College of Animal Science and Technology, Anhui Agricultural University, No. 130 Changjiang West Rd., Hefei 230036, China.
Tel.: +86 551 65786441
Fax: +86 551 65786441
E-mail: zhangzijun@ahau.edu.cn

List of Supporting Information

Table S1. Peptide Quality Statistics.

Figure S1. Experimental design and work flow for the integrative proteomics and transcriptomics comparison of high- and low-fecundity goat oviducts from Yunushang black goat in the follicular and luteal phases

Figure S2. Quality Control (QC) Analysis.

Figure S3. WGCNA Analysis.

Figure S4. Combined Analysis of Proteome and Transcriptome

Table S1 Peptide Quality Statistics

Sample	Peptides	Proteins
FH1	52398	5095
FH2	49555	5072
FH3	51265	5015
FH4	46004	4884
FH5	49614	5003
FL1	54122	5131
FL2	46096	4737
FL3	49045	5014
FL4	43199	4718
FL5	48981	5013
LH1	42825	4741
LH2	45790	4902
LH3	46926	4951
LH4	50134	4992
LH5	47397	4944
LL1	48880	4979
LL2	45955	4935
LL3	43430	4778
LL4	46617	4955
LL5	46307	4976

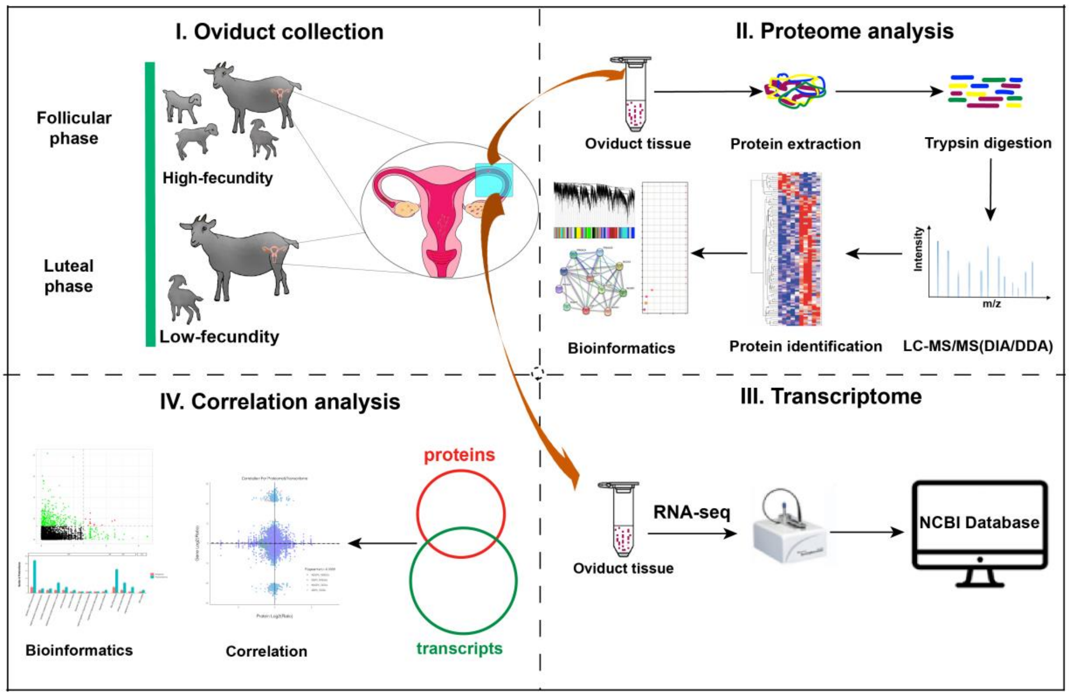

Figure S1. Experimental design and work flow for the integrative proteomics and transcriptomics comparison of high- and low-fecundity goat oviducts from Yunushang black goat in the follicular and luteal phases

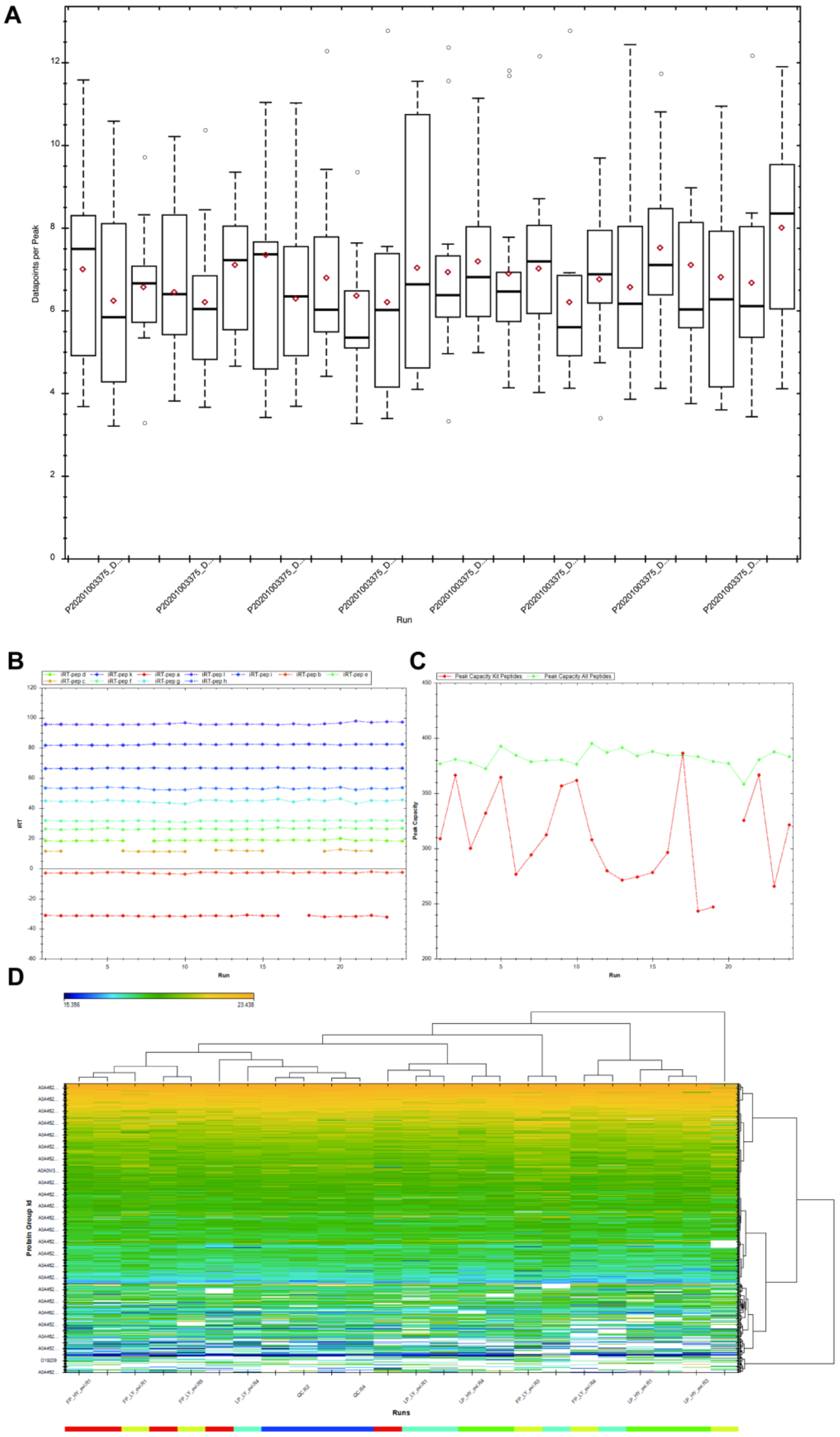

Figure S2. Quality control and quantitative heat map of DIA. (**A**) Average data points per peak: the average data points per peak were more than 5, which met the requirements of quantitative analysis. (**B**) Chart of iRT elution time: the main iRTs were detected and the retention time was generally stable. (**C**) Column peak capacity statistics: the abscissa was the order of the samples, the green line was the data of all peptides, and the red one was the data of the iRT internal standard. Peak capacity represented the separation and analysis capability of the column. The average peak capacity was more than 200, indicating better separation and analysis. (**D**) Quantitative heat map of DIA.

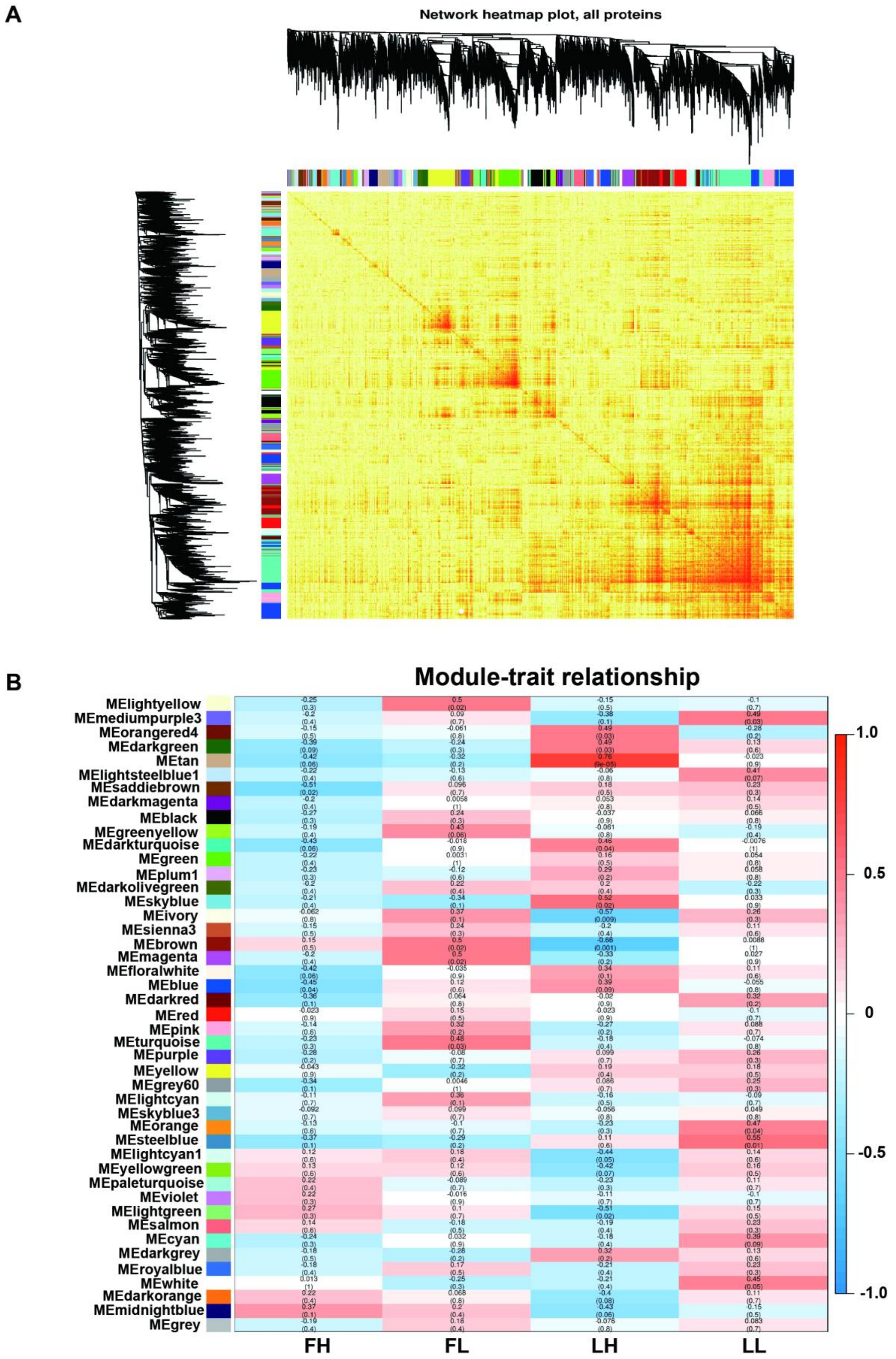

Figure S3. Weighted gene co-expression network analysis results. (**A**) Clustering dendrograms of proteins, with dissimilarity based on topological overlap with the assigned module colors. (B) Module-trait associations. Each row corresponds to a module, column to a trait attribute. Each cell contains the corresponding correlation and p value. The color bar on the right represents the magnitude of the correlation coefficient, with red representing positive correlation and blue representing negative correlation.

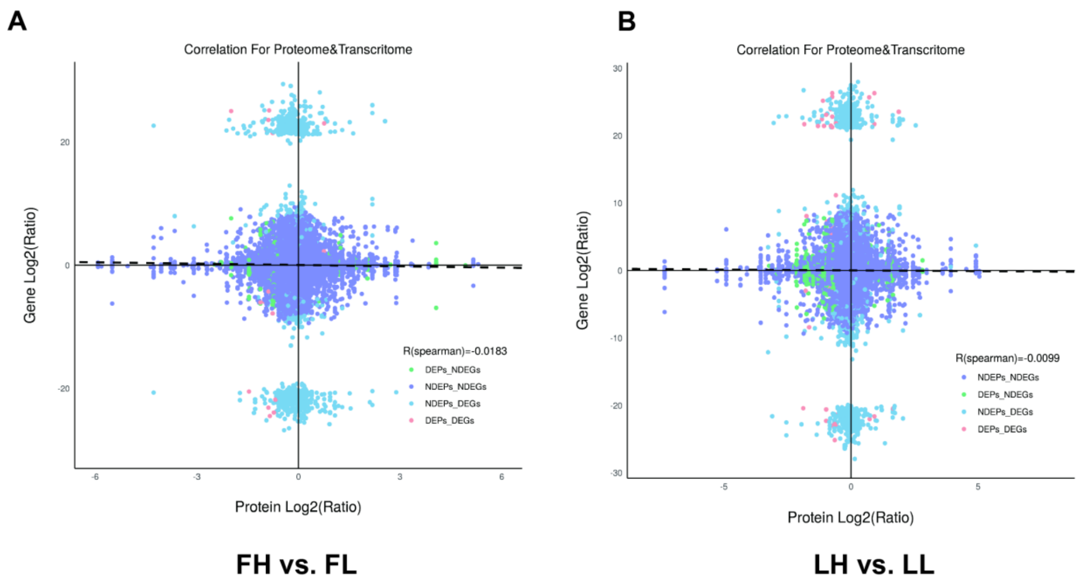

Figure S4. Comparison of abundance ratios from transcriptomic and proteomic profiling in the follicular**(A)** and luteal phases **(B)** . X-axis indicates that the fold difference in protein expression in each comparison group (log_2 Ratio); Y-axis indicates that the fold difference in gene expression in the comparison group (log_2 Ratio); Purple dots (NDEPs_NDEGs) indicate no significant difference for both genes and proteins, green dots (DEPs_NDEGs) indicate no significant difference for genes and significant difference for proteins, blue dots (NDEPs_DEGs) indicate significant difference for genes and no significant difference for proteins, and red dots (DEPs_DEGs) indicate significant difference for both genes and proteins.
